# Deciphering Neural Mechanisms Underlying Marmoset Dynamic Natural Behaviors Using a Miniaturized Wireless Large‐Scale Coverage Neural Recorder

**DOI:** 10.1002/advs.202507110

**Published:** 2025-10-27

**Authors:** Hongru Liu, Xinyuan Cao, Jiyong Li, Lingyi Zheng, Jingwei Li, Qianbing Li, Min Xie, Huimin Li, Xiaolong Wang, Yuyu Wu, Xiangyu Zhang, Yizheng Wang, Xize Gao, Tiancheng Sheng, Nianzhen Du, Chengao Xu, Kai Zhou, Jing Xu, Changxiang Yan, Lianqing Liu, Lixia Gao, Xinjian Li, Mingjun Zhang

**Affiliations:** ^1^ School of Biomedical Engineering Tsinghua University Beijing 100084 China; ^2^ Department of Neurology of the Second Affiliated Hospital and Interdisciplinary Institute of Neuroscience and Technology Zhejiang University School of Medicine Hangzhou 310029 China; ^3^ Key Laboratory of Biomedical Engineering of Ministry of Education, College of Biomedical Engineering and Instrument Science Zhejiang University Hangzhou 310027 China; ^4^ Department of Mechanical Engineering Tsinghua University Beijing 100084 China; ^5^ Department of Neurosurgery, Sanbo Brain Hospital Capital Medical University Beijing 100093 China; ^6^ State Key Laboratory of Robotics and Intelligent Systems, Shenyang Institute of Automation Chinese Academy of Sciences Shenyang 110016 China; ^7^ Nanhu Brain‐computer Interface Institute Hangzhou 311100 China; ^8^ NHC and CAMS Key Laboratory of Medical Neurobiology, MOE Frontier Science Center for Brain Science and Brain‐machine Integration, School of Brain Science and Brain Medicine Zhejiang University Hangzhou 310058 China

**Keywords:** freely moving neural recording, high‐density flexible µECoG array, neural dynamics underlying natural behaviors, non‐human primate, wireless neural recorder

## Abstract

Deciphering neural mechanisms underlying dynamic natural behaviors of freely moving species requires long‐term recordings of large‐scale brain activities. However, most conventional neural recorders are limited by their weights and measures, electrode coverage, and signal throughput, hindering the dissection of underlying neural mechanisms. This study reports real‐time large‐scale recordings and deciphering of brain activities from frontal and temporal cortices of freely moving marmoset across various natural behavioral repertoire using a miniaturized wireless neural recorder comprising a custom‐designed 120‐channel flexible µECoG array. Behavior‐specific highly resolved spatiotemporal neural dynamics are observed, including alpha‐band activations during drinking, anticipatory responses before vocalization, and transient high‐gamma increase during vigilance to human intruders. Three phases of drinking behavior are identified using multi‐area neural features captured by the recorder with an accuracy exceeding 87%. After over 16 months (March 13, 2024‐August 1, 2025, remaining actively recording) of recordings, the neural signals acquired using the recorder maintain high fidelity and low attenuation during both the resting and drinking states, enabling potential long‐term dissection of the neural mechanisms of natural behaviors in freely moving marmosets.

## Graphical abstract

This study introduces a miniaturized wireless neural recording system integrated with custom‐designed 120‐channel µECoG arrays, engineered for monitoring brain activities in freely behaving marmosets. The system can capture and characterize behavior‐specific neural signals, including drinking‐related activation, pre‐vocalization anticipatory responses, and vigilance‐induced transient responses, enabling advanced studies of neural mechanisms underlying natural primate behaviors.

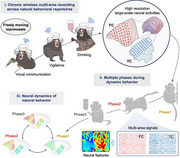

## 1 Introduction

Dynamic natural behaviors of freely moving species typically involve intricate interactions of large‐scale neural populations and coordinated actions across multiple brain regions.^[^ ^1^, ^2^, ^3^ ^]^ This is particularly evident in freely moving non‐human primates (NHPs), which leverage evolutionarily enhanced multi‐area neural coordination to support complicated behaviors, including social interactions, cognitive processes, and adaptive motor skills.^[^ ^4^, ^5^, ^6^, ^7^ ^]^ Long‐term recordings of large‐scale brain activities of NHPs may provide crucial insights into understanding their sophisticated neural mechanisms in perception, learning, and decision‐making.^[^ ^8^, ^9^, ^10^, ^11^ ^]^ The common marmoset, a small New World primate, demonstrates striking similarities to humans in innate behaviors, vocal communication, and emotional response,^[^ ^12^, ^13^, ^14^ ^]^ makes it an ideal model for investigating intricate neural mechanisms underlying natural behaviors.^[^ ^15^, ^16^, ^17^ ^]^

Common marmosets often exhibit rapid 3D locomotion and highly flexible limbs, making their natural behaviors exceptionally dynamic.^[^ ^18^ ^]^ Deciphering the neural mechanisms underlying these behaviors remains a daunting challenge in neuroscience research. Most studies in the open literature investigated neural activities in restricted or anesthetized marmosets using techniques such as single‐electrode recordings, functional magnetic resonance imaging (fMRI) and two‐photon calcium imaging.^[^ ^19^, ^20^, ^21^, ^22^, ^23^ ^]^ However, behavioral repertoires and neural dynamics in constrained states could differ significantly from those in freely moving states.^[^ ^24^, ^25^ ^]^ Therefore, neural monitoring during free movement is of great importance for understanding cognitive functions and interregional coordination underlying natural behaviors. Although multiple wireless electrophysiological recording systems for freely moving marmosets have been proposed,^[^ ^26^, ^27^, ^28^, ^29^ ^]^ they are confined to capturing neural activities from localized, highly constrained brain regions, lacking the capability for large‐scale mapping of coordinated neural dynamics across multiple brain regions involved in natural behaviors.

Several obstacles must be overcome in developing neural recording devices that can record large‐scale brain activities in real‐time across multiple brain regions in freely moving marmosets. First, the small body and light head size of marmosets make it difficult to carry a weighted device.^[^ ^14^, ^30^ ^]^ Furthermore, large‐scale monitoring of neural activities requires extensive electrode coverage. Achieving such coverage while minimizing surgical trauma presents significant challenges using conventional rigid deep brain electrodes.^[^ ^31^, ^32^, ^33^ ^]^ In addition, high electrode density and system sampling rates are essential for acquiring neural signals with accurate spatial and temporal resolution.^[^ ^34^, ^35^, ^36^ ^]^ However, this imposes constraints on the wireless signal transmission capabilities of a neural recording system. As a result, lightweight, wireless neural recording devices with compact size, extensive brain region coverage, and high wireless signal throughput are highly anticipated for studying natural behaviors in marmosets.

Here, we report an integrated solution for dissecting the underlying neural mechanisms across various natural behavioral repertoires using a miniaturized wireless neural recorder. The recorder is featured with i) a 120‐channel real‐time recording and transmission wireless device; ii) lightweight, miniaturized modular assembly to minimize disruption of natural behaviors; iii) two custom‐designed, high‐density 60‐channel flexible µECoG arrays tailored to the morphology of the targeted brain areas. The µECoG arrays were implanted in the frontal cortex (FC) and temporal cortex (TC). The FC array facilitates the study of motor function, high cognitive functions, decision‐making processes, and goal‐directed behaviors,^[^ ^37^, ^38^ ^]^ while the TC array makes it possible to investigate auditory processing, sensory integration, and vocalization‐related neural circuits during social communication.^[^ ^39^, ^40^ ^]^

The neural recorder has been employed to acquire neural signals from multi‐brain regions under three natural behaviors of freely moving marmosets. We observed behavior‐specific highly resolved spatiotemporal neural activities, including alpha‐band activation during drinking, anticipatory responses before vocalization, and transient high‐gamma increase during vigilance to human intruders. The decoding of different drinking phases reaches an accuracy exceeding 87%. Chronic recordings in both resting and drinking states demonstrate the reliability and stability of the recorder for extended observation of large‐scale brain activities. The modular system has demonstrated its capability for real‐time large‐scale recordings and deciphering of brain activities, which may pave an avenue for understanding complex cognitive functions and neural mechanisms of various behaviors in freely moving objects.

## 2 Results

### 2.1 Overview of the Neuro‐Behavioral Research Procedure Using a Customized Neural Recorder in Freely Moving Marmosets

**Figure 1:**
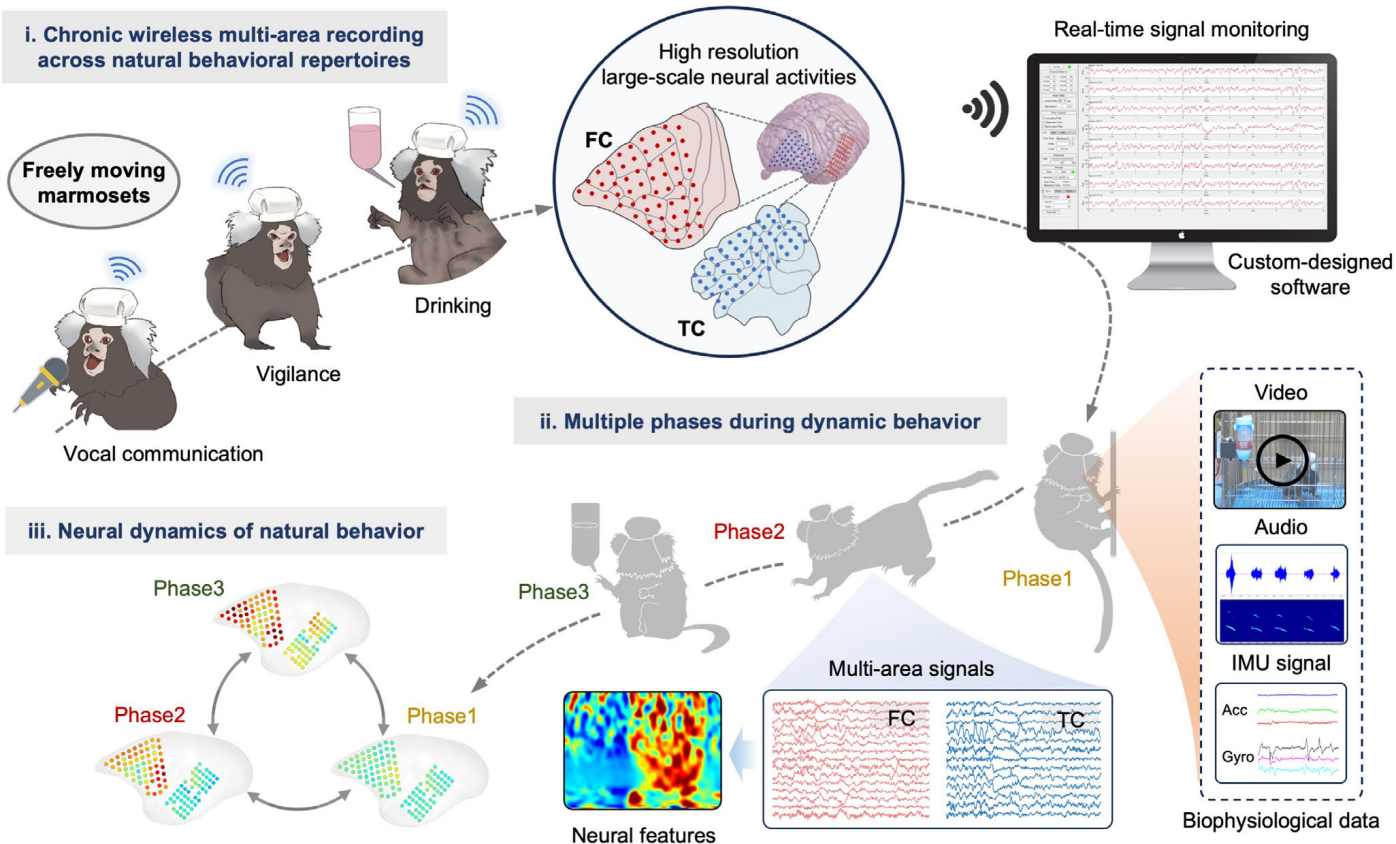
Neuro‐behavioral research underlying natural behaviors in marmoset using a customized neural recorder. The diagram shows the neurobehavioral research flow of a marmoset's natural behaviors including three parts: i) The freely moving natural behaviors in this study include drinking, vocal communication, and vigilance to human intruders. Behavior‐related neural signals of large‐scale brain regions are recorded using the neural recorder and transmitted to the upper computer in real‐time. ii) Acquired multi‐area neural signals are offline synchronized with multiple phases of dynamic behavior and converted into scalograms for time‐frequency representation. iii) The spatiotemporal neural dynamics of multiple frequency bands across multi‐brain regions are presented during their natural behaviors. IMU, inertial measurement unit. ACC, accelerometer. GYRO, gyroscope.

To decipher neural mechanisms underlying the dynamic natural behaviors of marmosets, it is required to record neural activities in real‐time across multiple brain regions in freely moving marmosets. Taking into consideration of the limitations of the previous methods, we present here an integrated solution to dissect the neural mechanisms of various natural behavioral repertoires. The detailed pipeline includes three parts (**Figure** **1**). Specifically, after the marmoset recovered from the surgery and adapted to the customized device, we recorded the µECoG signals of the FC and TC regions in real‐time using our custom‐designed upper computer software when the marmoset performed various behaviors including locomotion, innate reward, communication‐related vocalization and vigilance response to human intruders. Simultaneously, the animals’ behaviors including locomotion, and acoustic response are recorded to identify different phases of ongoing behavioral activities. The µECoG signals and marmoset's behaviors are offline synchronized with diverse phases and the µECoG signals are transformed into scalograms for time‐frequency representation, which allows us to analyze the highly resolved spatiotemporal patterns of neural activities. Finally, the processed signals are utilized as distinguishable neural features to construct and train a neural decoder, which was used to classify the phases of various natural behaviors. This approach enables the extraction and analysis of neurophysiological data collected directly from the brain during the animal's natural behaviors, providing integrative, scientific and objective insights into the brain neurobehavioral processes.

### 2.2 Custom‐Designed, Miniaturized, Multi‐Area Coverage, Real‐Time Wireless Neural Recording Platform

**Figure 2:**
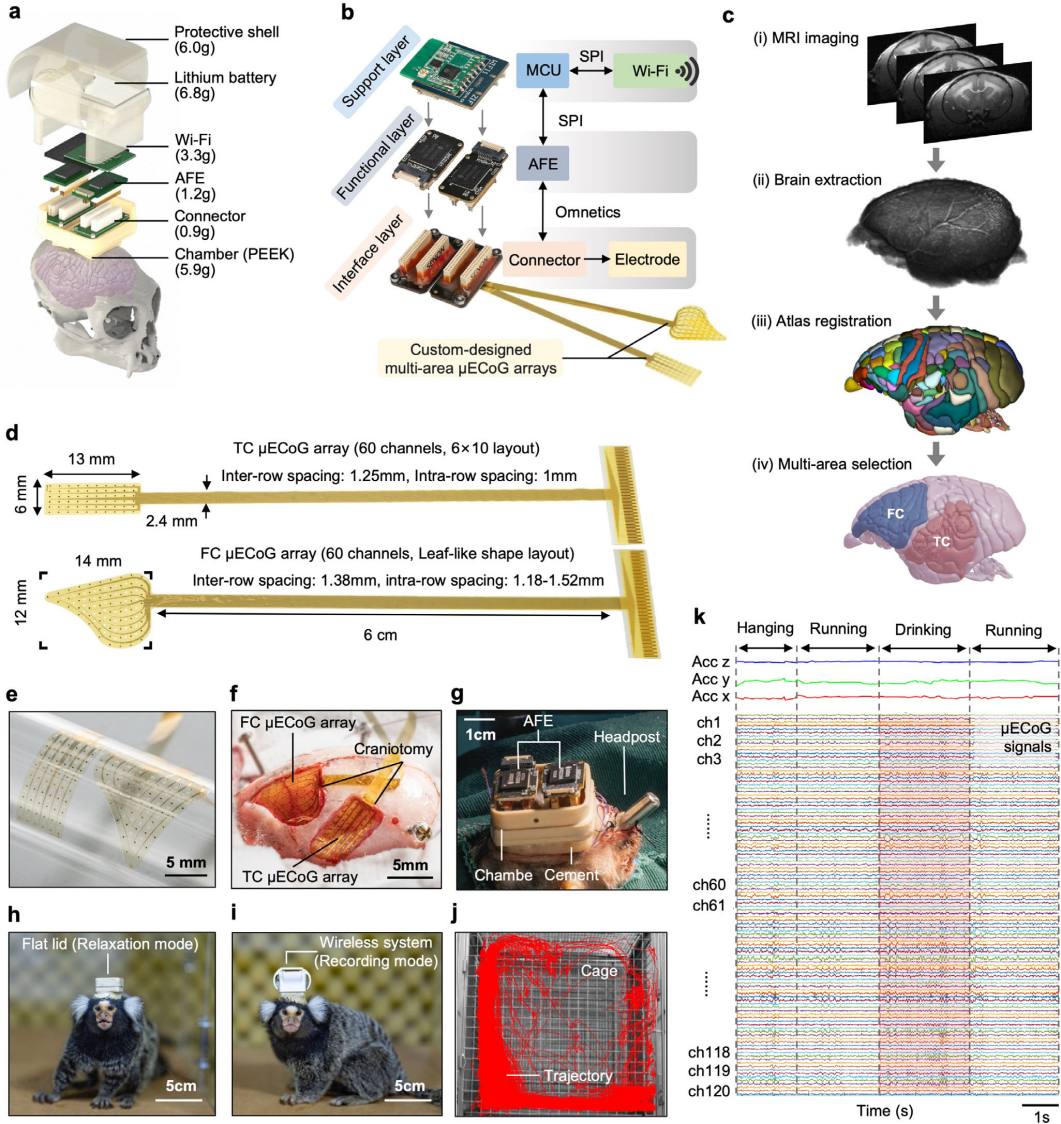
Overview of the miniaturized multi‐area coverage, real‐time wireless neural recording platform. a) Exploded view of the neural recording platform. The weights of different assemblies are indicated below. b) The neural recorder consists of three stacked layers: support layer, functional layer, and interface layer. The interface layer can be adapted to different custom‐designed electrode arrays to target various brain regions based on experimental design. c) Key steps to custom‐design flexible µECoG arrays tailored to the morphology of the targeted brain areas. d) Schematic diagram showing the two µECoG arrays. Left, implanted part. Right, input‐output part. e) Implanted part of the two µECoG arrays. The morphologies were designed based on the targeted cortical regions in the marmoset brain. f) Schematic diagram showing the two µECoG arrays that covering the FC (Upper) and TC (Bottom). g) The platform was installed inside a 3D‐printed PEEK chamber, which was implanted on the head of the marmoset. h,i) Diagram showing the head‐attached neural recording system during relaxation (h) and recording (i). j) The locomotion trajectory of the marmoset, which carried a head‐attached neural recording system in the homecage for 2 h. k) A represented image showing the 120‐channel neural signals and 3‐axis accelerometer signals during freely moving behaviors.

In present study, we have designed a miniaturized, multi‐area coverage, real‐time wireless neural recording platform. The platform comprises a modular miniaturized wireless system, paired with two custom‐designed high‐density 60‐channel flexible µECoG electrode arrays packaged in a plug‐and‐play headstage. **Figure** **2**a presents the heterogeneous architecture of the headstage (Figure 2a). The compact and three‐layer modular design (Figure 2b) integrates a support module for 120‐channel wireless communications with upper computer, two custom‐designed 60‐channel Analog Front Ends (AFEs), and adaptors to connect the µECoG electrodes. The entire headstage weighs ≈26.2 g and is housed in a 3D‐printed polyether ether ketone (PEEK) chamber (5.9 g with dimensions of 25 x 27 x 15 mm), allowing the device to be securely attached to the marmoset's skull for extended periods of time. The plug‐and‐play modular design allows the weight of the headstage to be reduced to 13.4 g (relaxation mode) by removing the support layer and battery, when not in use.

Previously used µECoG arrays usually cover a large part of the brain regions, which requires removal of the above skulls during surgery and could increase the risk of surgical trauma. To minimize the surgical damage, we tailored the µECoG electrode outlines to match the morphology of the target brains, including FC and TC, using marmoset MRI brain imaging (Figure 2c). Based on the morphology of FC and TC, the µECoG arrays are custom‐designed to be a leaf shape in the FC and rectangle in TC (Figure 2d). The electrodes within these regions are evenly distributed, and the inter‐site distance of the electrodes is 1 mm (Figure 2d). Meanwhile, the µECoG arrays have a thickness of 4 µm and were patterned with uniformly distributed perforations holes (300 µm in diameter, Figure S6a,b, Supporting Information) to allow cerebrospinal fluid drainage from the electrode contact sites.^[^ ^41^ ^]^ Notably, the ultrathin structure combined with the perforation design enables large‐area µECoG arrays to conformally adhere to the cortical surface (Figure 2e,f; Figure S6c,d, Supporting Information). Considering that the µECoG arrays are designed for long‐term neural recordings, their mechanical reliability is also crucial. Repeated bending tests (100 cycles, Figure S8, Supporting Information) revealed a negligible increase in electrode impedance (≈ 6.4%, from 202 to 215 kΩ), demonstrating their robustness under the mechanical stress. The custom‐designed flexible µECoG electrode arrays were implanted on the brain surface of one marmoset, and the neural recording platform was installed on the skull using screws and dental cement (Figure 2f,g). After the marmoset recovery from the surgery, the neural activities in multiple brain regions, together with animal behaviors were recorded simultaneously (Figure 2h–k). To test whether the implants impaired the animal's locomotion, we compared the locomotor behavior of the marmoset with and without head implants in the homecage for 2 h. It was found that the marmosets with the device implants exhibited similar or even better locomotion behaviors including hanging time, movement distance, and jump numbers (Figure S10, Supporting Information). Thus, our flexible and high‐density µECoG arrays together with the proposed neural recorder in the present study provide a solution to real‐time monitor the large‐scale neural activities of marmosets during high‐dynamic natural behaviors.

### 2.3 Distinct Spatial Neural Activities During Drinking in both FC and TC

**Figure 3:**
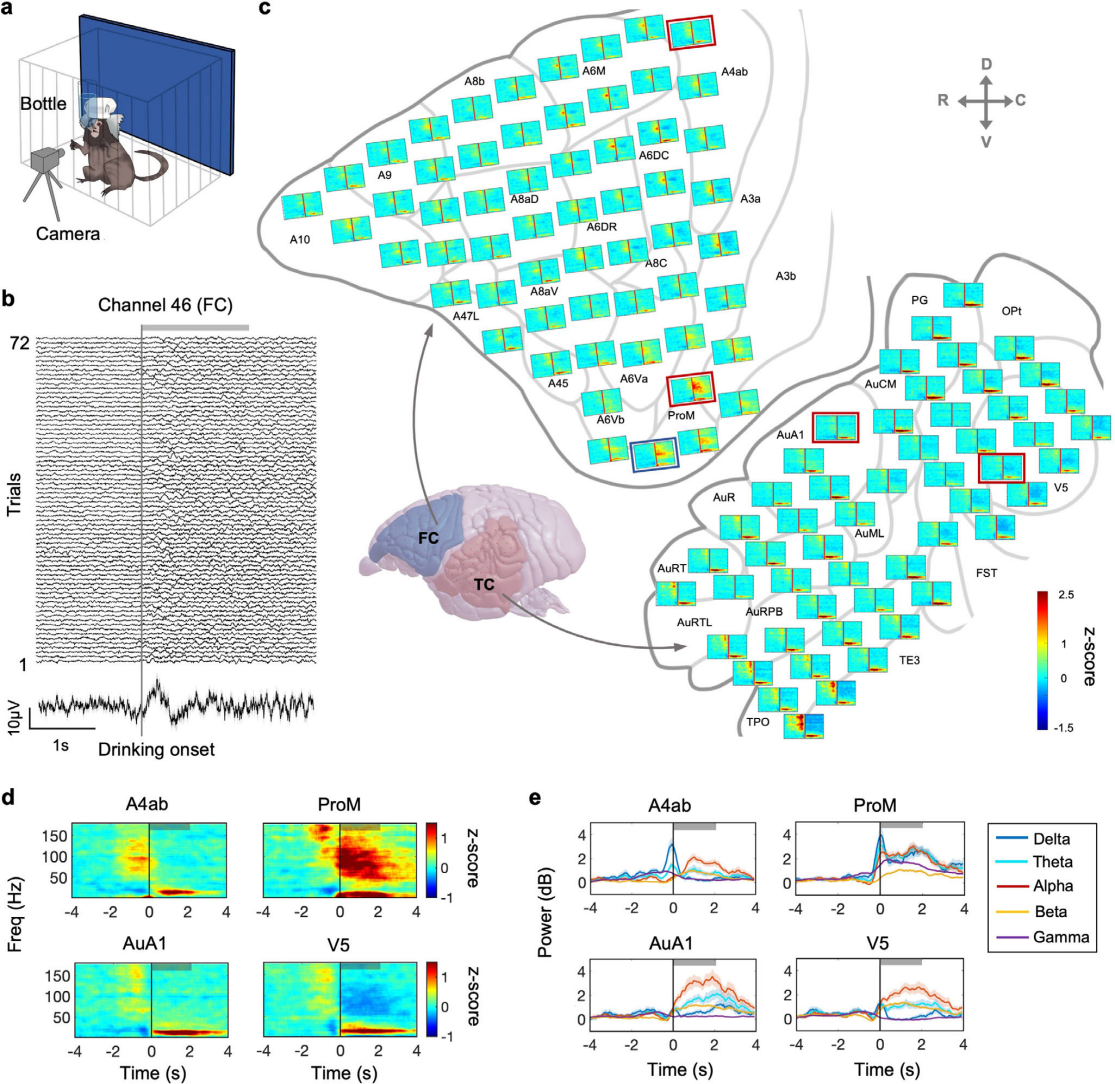
Distinct highly resolved neural activities during drinking in both FC and TC. a) Schematic image showing the experimental setup of the drinking behavior. The behavior arena includes 1 test cage (size: 0.5m x 0.5m x 0.4 m) and 1 video camera. A water bottle hanging on the cage was used to freely deliver juice reward. The frontal‐view camera was used to capture the locomotion and drinking behaviors. One marmoset was placed in the marmoset cage and could freely access the reward for ≈ 60 min. Meanwhile, the neural signals in both TC and FC with the drinking behavior were recorded. b) Sampled images showing the drinking‐induced µECoG signals of ProM region (Channel 46, blue border in (c)) at single trial (*n* = 72 trials) or trial averaged level (mean ± SEM). The neural signals were aligned to the drinking onset. Thick gray bar: average drinking duration for 2.1 s. c) Schematic image showing the trial‐averaged spectrograms of drinking‐induced neural activities using both FC and TC µECoG arrays (*n* = 72 trials). 10 damaged and high‐impedance channels were discarded and displayed as empty. d) Spectrograms of four sampled electrodes from 4 separated brain regions (A4ab, ProM, AuA1, V5, red border in (c)). Vertical line: the drinking onset. Thick gray bar: average drinking duration. e) The sampled neural responses of A4ab, ProM, AuA1, V5 during drinking at five frequency bands. The power from each frequency band was aligned following the drinking onset and averaged across trials (mean ± SEM). Blue: Delta band, 1‐4 Hz; Cyan: Theta band, 5–8 Hz; Red: Alpha band, 9–14 Hz; Yellow: Beta band, 15–30 Hz; Purple: Gamma band, 31–180 Hz.

Three months after the electrode‐implant surgery, we recorded the neural activities of FC and TC while the marmoset freely accessed water reward in the homecage (**Figure** **3**a). The neural signals in one example channel were aligned to marmoset drinking onset and averaged across trials (Figure 3b). Clearly, the example channel displayed reliable dynamic neural activities at single‐trial level during drinking (Figure 3b), indicating high stability of the neural signals recorded by the wireless neural recorder. Then, the neural signal in each channel was decomposed to spectral and temporal components using short‐time Fourier transform (STFT) and the spectrograms were averaged across trials. The spectrograms in each channel were mapped to the surface of the FC and TC (Figure 3c). We found distinct drinking‐related neural activities distribution patterns between FC and TC (Figure 3c). Specifically, we used four example channels to display the brain region‐specific drinking activities including sensory‐motor (ProM, A4ab), auditory (AuA1), and high‐order vision regions (V5) (Figure 3d,e). In addition, we calculated the averaged power of µECoG signals from five representative bands (delta, theta, alpha, beta, and gamma) to display the drinking‐related neural activities. As shown in Figure 3d,e, low‐frequency signals (1‐30 Hz) were widely distributed across nearly all brain regions during the drinking (Figure 3d,e). Interestingly, high‐frequency signals (gamma band) during drinking were observed to increase in the parietal region (ProM), but to decrease in the high‐order vision region (V5), indicating the ProM may dynamically respond or encode the mouth‐related movement (Figure 3d,e, right top). So FC and TC displayed distinct spatial neural activities during drinking.

### 2.4 Classifying Different Phases of Water Drinking Using Neural Activities from Both FC and TC

**Figure 4:**
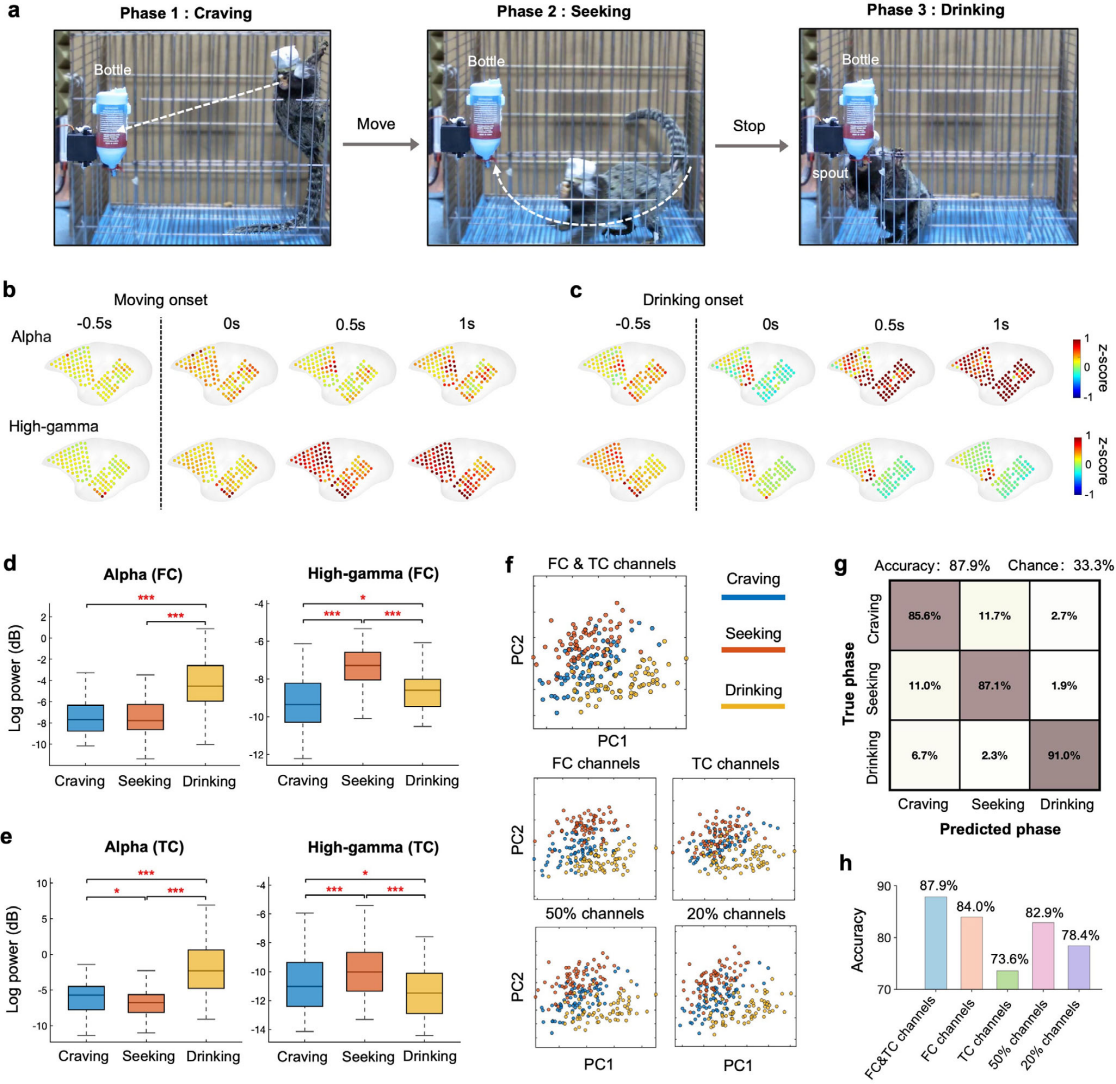
Decoding three phases of the drinking behaviors using global FC and TC activities. a) Representative behavior images showing three phases (craving, seeking, and drinking) of water reward behaviors. b,c), Spatial distribution of two neural activities bands (alpha and high‐gamma) power across FC and TC from water craving, seeking to drinking (*n* = 72 trials). The neural signals were aligned separately by the movement onset in (b) and the drinking onset in (c). d,e) Averaged power of alpha and high‐gamma bands for all the FC and TC electrodes during the three phases of water drinking (*n* = 72 trials). Wilcoxon signed‐rank test (*P < 0.05, **P < 0.01, ***P < 0.001). f) State‐space analysis using principal component analysis (PCA) showed the separation of clusters for the three phases. Each trial of the three phases was represented in the 2D state‐space by the first two principal component scores (*n* = 72 trials). g) Confusion matrix for the three phases using linear support vector machine method (SVM) decoding demonstrated high accuracy for each phase. h) Classification accuracies for different sub‐sampling strategies and the number of brain regions.

The water‐drinking behaviors in our task include three phases: craving, seeking, and drinking^[^ ^42^ ^]^ (**Figure** **4**a). The craving phase is between the marmoset visualization of the water bottle and the animal begins to move toward the water bottle. The seeking phase is the time that the animal moves to the water bottle. The drinking phase is the time window during which the animal takes the juice reward (Figure 4a). Three phases indicate the animal's three different behavior states and may induce distinct neural activities. To answer this question, we mapped the neural activities of different bands across these three phases on the surface of FC and TC (Figure 4b,c). Here we focus on the alpha and high‐gamma bands that highly correlated with the behaviors. Notably, the alpha‐band power in the drinking phase was significantly elevated compared to the craving and seeking phases, especially on TC (Figure 4d,e). Instead, the FC region showed a higher high‐gamma band during seeking phase indicating the water anticipation signal before the drinking phase (Figure 4d,e).

To examine similarity of the neuronal activities in different phases of drinking behaviors, we analyzed the FC and TC's activities with principal component analysis (PCA), which transforms covarying multi‐band features across different phases and trials into low‐dimensional representations (Figure 4f) (see Experimental Section). When using signals from all the FC and TC channels, visualization of the principal component scores in a 2D vector space revealed distinct separations of different phases (Figure 4f). To further evaluate the impact of electrode spatial resolution and coverage on cortical state‐space representation, we repeated the analysis using spatially subsampled electrodes and reduced brain region coverage. The reduced spatial resolution and limited coverage resulted in diminished separability of different phases, highlighting advantages of high‐density multi‐area µECoG arrays. In addition, based on the multi‐band neural features reduced using the PCA technique, we employed a 5‐fold cross‐validated support vector machine (SVM) to classify the three phases for drinking (see Experimental Section). Leveraging neural features from all channels, the model achieved an average accuracy of 87.9%, demonstrating robust decoding performance across different phases (Figure 4g). We then varied electrode spatial resolution and selected regions to assess their impacts on decoding performance. Signals from FC contributed the most significantly to phase decoding, and a progressive decline in decoding accuracy was observed as spatial resolution decreased (Figure 4h). These results demonstrate that the high‐resolution multi‐area neural signatures captured by the recorder facilitate the phase classification of the drinking behavior.

### 2.5 A Higher Alpha Band was Found in FC Before and During Phee Production

**Figure 5:**
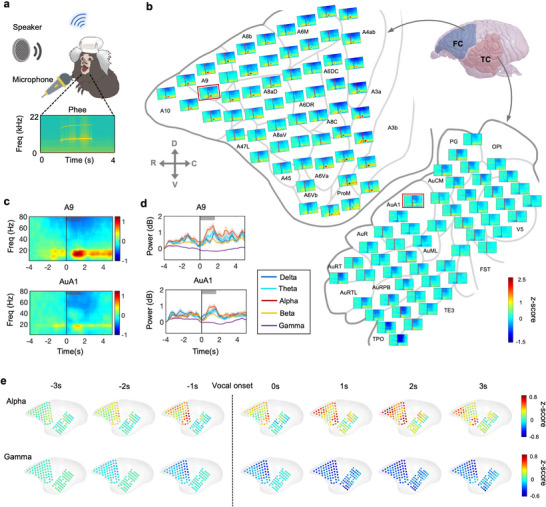
A higher alpha‐band was found in FC before and during a marmoset's phee production. a) Upper: Schematic image showing the marmoset vocal behaviors during neural activities recording using the µECoG arrays. The conspecific calls were played back to 1 freely moving marmoset to increase emitted calls during recording. Bottom: the spectrograms of 1 represented Phee call. b) Schematic image showing the trial‐averaged spectrograms using µECoG arrays on FC and TC during the marmoset emit phee calls (*n* = 214 trials). c) Spectrograms of two sampled electrodes from FC (A9) and TC (AuA1). Vertical line, the vocal onset. Thick gray bar: average phee call duration for 1.7 s. d) the sampled neural responses of FC and TC during the marmoset emitted phee calls at delta, theta, alpha, beta, and gamma bands (*n* = 214 trials). Blue: Delta band, 1–4 Hz; Cyan: Theta band, 5–8 Hz; Red: Alpha band, 9–14 Hz; Yellow: Beta band, 15–30 Hz; Purple: Gamma band, 31–80 Hz. e) Spatial distribution of alpha and gamma band across FC and TC during marmoset emitted phee calls.

Next, we recorded FC and TC's activities using µECoG arrays during the marmoset emitted calls (**Figure** **5**a). To increase emitted calls during recording, the conspecific calls were played back to one freely moving marmoset (Figure 5a). In this condition, the marmoset mostly emitted phee calls that have enough trails in recordings for further analysis (Figure 5a). Then we mapped spectrograms of the phee‐related activities on the surface of FC and TC (Figure 5b). We found several stronger activated brain regions including prefrontal regions (A10, A9), motor‐related regions (A6 and ProM) (Figure 5b). Then we used two represent electrodes in FC (A9) and TC (AuA1) to display phee‐related activities (Figure 5c,d). In addition, previous studies indicate that the alpha‐band is correlated with vocal communication, so we focus on the analysis of the alpha‐band during phee production.^[^ ^43^, ^44^ ^]^ Interestingly, the alpha‐band power in motor‐related regions and the prefrontal cortex increased by ≈1.2 s before the phee production, then decreased at the onset of the phee calls, and subsequently increased by 0.5 s after the phee production (Figure 5c–e). So motor‐related regions may carry both top‐down anticipation signals and motor‐related signals. However, the increased alpha‐band power in TC was only observed ≈0.5 s after vocal onset and was significantly weaker than that in FC (Figure 5c–e). Globally, the increased alpha‐band power was found ≈1.2 s before the phee production and ≈0.5 s after the phee production in the prefrontal cortex and motor‐related brain regions (Figure 5e). In contrast, gamma‐band (30–80 Hz) power was significantly suppressed in nearly all channels of TC and in a few channels of FC following vocal onset (Figure 5e; Figure S15a,c, Supporting Information). These findings suggest that vocal communication involves distinct pre‐vocal planning and post‐vocal adjustments across multiple brain regions.

### 2.6 Transient High‐Gamma Band Activation During Vigilance to Human Intruders

**Figure 6:**
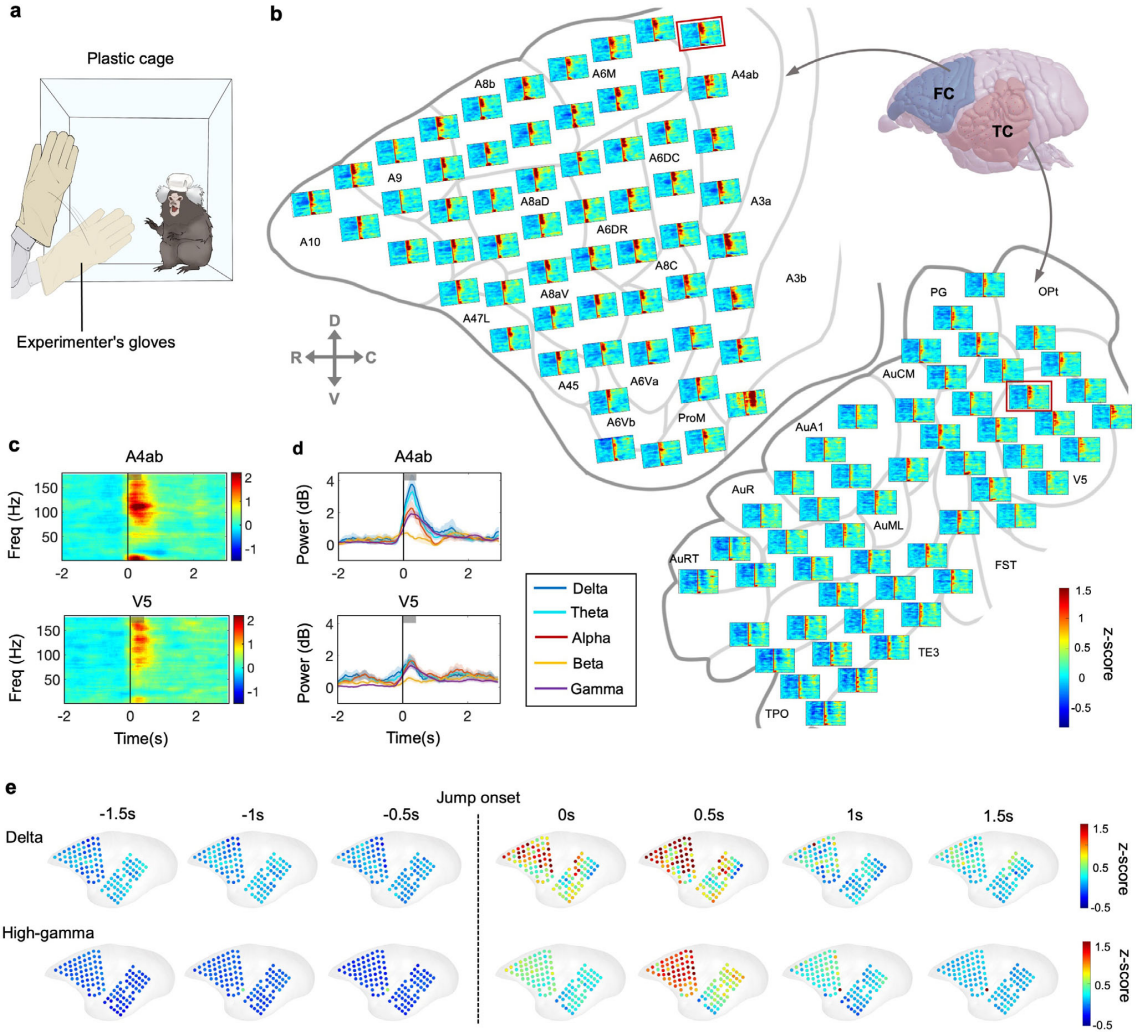
Transient high‐gamma activation during vigilance to human intruders. a) Schematic image showing the vigilance behavior when the marmoset visualized human intruders during neural activities recording using the µECoG arrays. The marmoset was placed in a custom‐designed cubic box in which only one side of the box was transparent. An experimenter swung a glove from the opaque side to the transparent side. Marmoset showed startled behavior and attempted to escape from the intruder. b) Schematic image showing the trial‐averaged spectrograms using µECoG arrays on FC and TC during the marmoset displays vigilance to human intruders (*n* = 40 trials). c) Spectrograms of two sampled electrodes from FC (A4ab) and TC (V5). Vertical line, the marmoset shows the escape jumping behavior. Thick gray bar: average jumping duration for 0.4 s. d) The trial‐averaged neural response of FC (A4ab) and TC (V5) during the marmoset shows the escape behavior at delta, theta, alpha, beta, and gamma bands (*n* = 40 trials). Blue: Delta band, 1–4 Hz; Cyan: Theta band, 5–8 Hz; Red: Alpha band, 9–14 Hz; Yellow: Beta band, 15–30 Hz; Purple: Gamma band, 31–180 Hz. e) Spatial distribution delta and high‐gamma band of FC and TC during the marmoset shows the escape jumping behavior.

Last, to understand the neural mechanisms of threatening and defensive behaviors in marmosets, we recorded the neural activities of FC and TC using our neural recorder when the marmoset displayed vigilance to human intruders. In details, the marmoset was placed in a custom‐designed cubic box in which only one side of the box was transparent. An experimenter swung a glove from the opaque side to the transparent side. The marmoset suddenly jumped and intended to escape from the intruder (**Figure** **6**a). Then, we mapped spectrograms of the vigilance‐related activities on the surface of FC and TC (Figure 6b). We found a transient high‐gamma activation after the marmoset started to escape from human intruders in almost all channels of FC and TC (Figure 6b). We similarly used two representative electrodes in FC and TC to display vigilance‐related activities (Figure 6c,d). After the onset of jumping, a rapid increase of multiple frequency band was found in both channels of FC and TC, lasting for ≈0.5 s (Figure 6c,d). Globally, the time of high‐gamma activation was delayed relative the delta band (Figure 6e). In addition, the responses in FC were stronger than those in TC (Figure S16a,b, Supporting Information). The above experimental results indicated that the vigilance‐related activities were broadly, but not evenly distributed in most regions recorded.

### 2.7 Long‐Term Stability of Multi‐Area Neural Signals During Both the Resting and Drinking States

**Figure 7:**
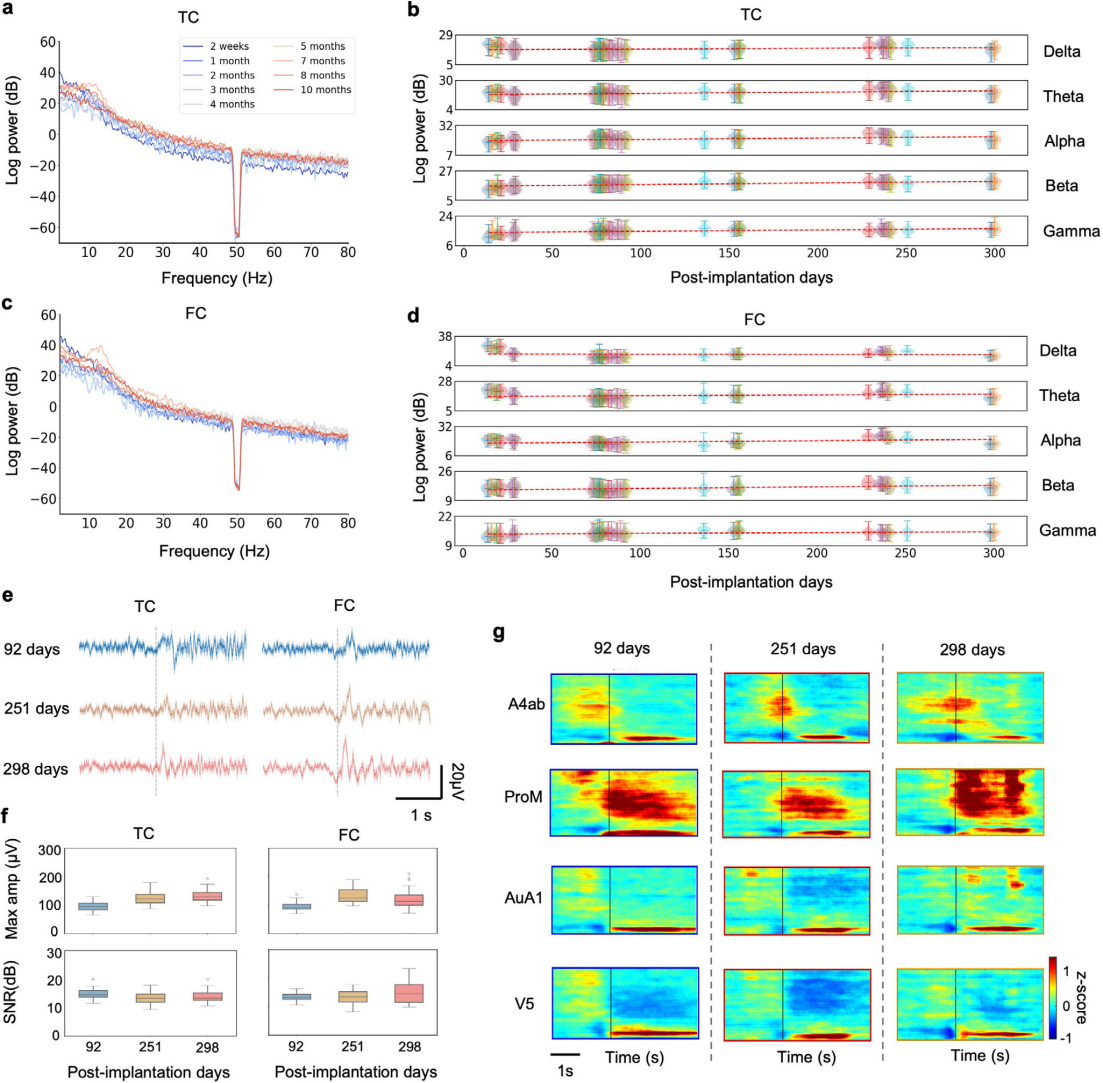
Long‐term stability of neural signals during both resting and drinking states. a,c) Power spectral density of signals recorded during the resting state from two representative channels from the TC and FC µECoG arrays over 10 months. b,d) Power variations of five typical band signals (delta: 1–4 Hz, theta: 5–8 Hz, alpha: 9–14 Hz, beta: 15–30 Hz, gamma: 31–150 Hz) recorded from all functional TC and FC channels (*n* = 110 channels) in resting state over 10 months. e) Waveforms of the neural responses (mean ± SEM) from two representative channels during drinking behavior at post‐implantation days 92 (*n* = 31 trials), 251 (*n* = 38 trials) and 298 (*n* = 34 trials). f) Maximum waveform amplitude (Max amp) and SNR of the neural responses during drinking behavior from the channels and days same as (e). g) Spectrograms from four channels representing four typical brain regions (A4ab, ProM, AuA1, V5) during drinking behavior at days same as (e). Vertical line: time zero of the drinking onset.

To measure long‐term stability of the acquired neural signals acquired from the neural recorder, we analyzed energy attenuation, response waveform, and signal‐to‐noise ratio (SNR) of the multi‐area signals in marmosets during both the resting and drinking states. In the resting state recordings, the marmoset was restrained in a chair to prevent interference with neural signals from scratching or movement. The power spectral densities (PSD) of signals from two representative channels in FC and TC exhibited minimal fluctuations over several months (**Figure** **7**a,c). Averaged power of five typical bands (delta, theta, alpha, beta, and gamma) of the resting state signals remained at similar levels from 14 to 300 days after the device implantation (Figure 7b,d).

To further investigate long‐term changes of multi‐area dynamics during drinking behavior, we analyzed the responses at three time points (92, 251, and 298 days after the device implantation). Figure 7e,f shows no significant changes in the waveform (Figure 7e) and SNR (Figure 7f) of the signals from two sampled channels in FC and TC. The averaged SNR at all time points was above 13.3 dB, with differences between averages not exceeding 1.8 dB (see Experimental Section). In addition, spectrograms of four typical regions (A4ab, ProM, AuA1, V5) maintained their distinct spatiotemporal neural patterns over time (Figure 7g). Overall, the neural signals over months maintained high fidelity and low attenuation, which demonstrated that the neural recorder can facilitate long‐term studies of natural behaviors.

## 3 Discussion

In this paper, we proposed a compact solution for real‐time large‐scale recordings and deciphering of brain activities underlying dynamic natural behaviors of freely moving marmosets using a miniaturized wireless neural recorder. Different from existing neural recording platforms^[^ ^21^, ^22^, ^27^, ^28^ ^]^ reported in the open literature for marmosets, the proposed neural recorder targets applications in deciphering neural mechanisms underlying nature dynamic behavior featured with custom‐designed electrodes, wireless recording, high throughput, large‐scale brain region coverage, and long‐term stability (Table S1, Supporting Information). Specifically, the high‐density µECoG arrays covered nearly the entire FC and TC, providing larger coverage than the excellent platforms reported the literature based on Utah arrays,^[^ ^28^ ^]^ silicon probes,^[^ ^21^ ^]^ or microwire electrodes.^[^ ^27^ ^]^ This extensive coverage is useful for investigating multi‐area neural dynamics. Compared to other ECoG electrodes for marmosets,^[^ ^22^ ^]^ the proposed µECoG arrays have the smallest inter‐electrode spacing, representing the high spatial resolution of ECoG signals. Furthermore, our electrode customization procedure employs a semi‐automated workflow based on established neuroimaging tools, which can be flexibly adapted to target any brain regions of interest. This scalable approach maintains the high spatial resolution advantages of our µECoG arrays, while enabling customized coverage for different experimental requirements. The results of decoding different drinking phases (craving, seeking, and drinking) indicated signals from a wider range of brain regions and higher spatial resolution facilitated the acquisition of distinguishable neural features and improved decoding performance. In addition, capacity for the 120‐channel real‐time wireless recording represents a high throughput currently achieved in marmosets.^[^ ^27^, ^28^ ^]^ The long‐term stability, demonstrated by over 16 months (March 13, 2024‐August 1, 2025, remaining actively recording) of high‐fidelity recordings, facilitates chronic studies examining neural plasticity and the influence of environmental factors on neural functions.

We revealed distinct spatiotemporal neural dynamics in FC and TC associated with drinking, vocal communication, and vigilance. During three phases of drinking behavior (craving, seeking, drinking), both the FC and TC showed enhanced alpha activities during drinking phase, indicated a distributed neural network underlying the motor control and execution of this goal‐directed behavior. One previous study of macaques demonstrated a similar increase in alpha/beta coherence between the primate anterior cingulate cortex gyrus (ACCg) and the basolateral amygdala (BLA) in a water‐reward experiment,^[^ ^45^ ^]^ which is consistent with our findings. Next, in phee calls production, we observed anticipatory alpha‐band activation in FC preceding vocal onset and post‐vocal gamma suppression in TC. The result is similar to previous microelectrode studies of the premotor cortex (PMC) and auditory cortical (AC) in marmosets during vocal production.^[^ ^29^, ^43^, ^44^, ^46^, ^47^ ^]^ However, we simultaneously monitored cortical signals from more brain regions compared to these studies, facilitating the understanding of the cortical auditory‐vocal network in marmosets. These findings suggest a crucial role of FC and TC in planning and executing vocalizations. Finally, in the human intruder experiments, the vigilance responses evoked a transient burst of high‐gamma activities, predominantly in FC. Gamma oscillations regulate the efficacious propagation of neural activities between cortical and subcortical regions.^[^ ^48^ ^]^ Such enhanced gamma oscillations were observed previously in multiple fear conditioning studies in rodents.^[^ ^49^ ^]^ This finding of a rapid high‐gamma increase in marmosets suggests immediate activation of a reactive mechanism essential for threat detection and the initiation of protective behaviors.

Despite the promising results obtained using the neural recorder, several limitations remain. The solution is primarily for recording and is currently not suitable for closed‐loop control of natural behaviors. Future developments will integrate stimulation functions along with more advanced analysis and decoding techniques, to investigate modulation of natural behaviors. NHPs have similar brain structures and neuronal functions to control advanced social, cognitive, and motor behaviors.^[^ ^8^, ^50^, ^51^, ^52^ ^]^ These findings in marmosets have translational potential for understanding and treating human neurological disorders. Our compact solution allows for long‐term collection of high‐resolution large‐scale brain activities from freely moving subjects, providing valuable opportunities for researching conditions characterized by motor impairments (e.g., Parkinson's disease), communication deficits (e.g., aphasia), and heightened reactivity (e.g., anxiety disorders).

## 4 Experimental Section

### Animals

Two adult marmosets (AZ and BQ) of either sex, at 1.5–2.5 years old of ages underwent surgery in this study (Table S2, Supporting Information). Two more marmosets (190 088 and BF) of either sex were used as the control group to compare the effect of surgery on the behavior of experiment animals. All experimental procedures were approved by Animal Use and Care Committee at Zhejiang University and in accordance with the National Institutes of Health Guidelines. The protocol number is ZJU20240803.

### Fabrication and Characterization of the Custom‐Designed µECoG Electrodes

The µECoG electrode arrays were fabricated on 4‐inch silicon wafers through conventional photolithography (Figure S4, Supporting Information). 1) A 2 µm thick polyimide film (HD‐2611, HD Microsystems) was formed on the substrate through spin‐coating and thermal curing. 2) A metal layer, Cr/Au (10 nm/100 nm), was patterned on the polyimide film via photolithography, physical vapor deposition, and lift‐off processes utilizing AR‐BR 5480 and AZ 601 photoresists, a manual mask aligner (URE‐2000/35, Chengdu Jingpu Technology Co., Ltd), and a magnetron sputtering system (JCP500, Beijing Technol Science., Co., Ltd). 3) Step 1 was repeated to form the top encapsulation layer of the electrode arrays. 4) The stacked layers were patterned with a layer of AZ4620 positive photoresist (6 µm) and dry etched with an inductively coupled plasma (ICP) etching equipment (NE‐550H, ULVAC) to expose the electrode recording sites, I/O pads, and define the shape of the electrode arrays. 5) The electrode arrays were peeled off from the substrate using a water‐soluble tape (Water‐soluble wave solder 5414, 3M), and immersed in deionized water at room temperature overnight to achieve complete dissolution of the water‐soluble tape. The released electrode arrays were assembled with the wireless neural recorder before use and sterilized using an ethylene oxide sterilization system (HM78, Tianjin SATOU Environmental Machinery Co., Ltd).

The electrochemical impedance spectroscopy (EIS) of the µECoG electrode arrays was measured in physiological saline at room temperature (Figure S5, Supporting Information), using a CHI660E electrochemical workstation (CHI660e, CH Instruments Inc., China). The measurement setup utilized a three‐electrode configuration, with an Ag/AgCl electrode as the reference electrode and a Pt wire as the counter electrode. In order to demonstrate the yield and manufacturing stability of the custom‐designed µECoG technology, impedance measurements were conducted for six sets of 120‐channel µECoG arrays (from three batches, two sets per batch, Figure S7, Supporting Information). The results showed that all six µECoG arrays achieved yields exceeding 90%, with their 1 kHz impedance values clustering within a narrow range of 136.5‐204 kΩ (median values), indicating both high manufacturing yield and stable electrical performance across all fabricated devices.

### MRI

7T MRI scanning were performed on the animal before the surgery, and coordinates of locations for implantation were calculated according to the MRI scanning result. During brain MRIs, alfaxalone (1 ml kg^−1^) was injected into muscle first as anesthesia induction, and the anesthesia state maintained by inhalation of isoflurane (0.8–1.5% in 100% oxygen). Marmoset was immobilized in a custom‐made stereotaxic apparatus. Water‐warmed blanket was used to maintain the body temperature of marmoset. The MRI experiment was conducted on a 7.0T MRI scanner (Siemens Healthcare). 3D coronal images were acquired with following parameters: 0.33 mm slice thickness, flip angle 7°, acquisition voxel size 0.3 × 0.3 × 0.3 mm.

### Surgical Procedure for Implanting the Flexible µECoG Electrodes and Headstage

Atropine (0.045 mg kg^−1^) was injected into muscle first to inhibit salivary secretion. Anesthesia was induced with an intramuscular injection of ketamine (40 mg kg^−1^) and maintained by inhalation of isoflurane (0.8–1.5% in 100% oxygen). Animals breathed spontaneous through tracheal intubation. Heart rate, respiration rate, blood oxygen saturation, and temperature of animal were monitored constantly during the entire surgery.

After shaving and disinfecting the skin, the animal was placed in a stereotaxic apparatus. The head was fixed securely. An incision was made along the midline of the head. Temporal muscles were peeled off the skull from the superior temporal line without resection. Outline the left lateral sulcus. Skull should be cleaning completely using hydrogen peroxide and saline. Contours of electrodes were outlined on the skull using template pieces with reference to the lateral sulcus and coordinates calculated before. The place where three bone screws and chamber would be fixed needed to be drawn as well. Cranial windows were drilled along the outline of electrodes and the removed bone flaps were preserved in saline solution carefully. Drilled three holes and screwed three bone screws as signal ground. Due to the thinness of marmoset skull, the length of bone screws inserted could not exceed 1.25 mm. Micro‐manipulation arm with chamber and electrodes fixed was used for electrode bonding. After µECoG electrodes placed in correct positions, replaced bone flaps in the original position and bound with mixture of dental cement and glue. Chamber was adhered on the skull, and dental cement was injected into the cavity of it to ensure the chamber was completely integrated with the skull. Bone screws and one headpost were also secured with dental cement. Protective shell was covered after the cement had hardened. The wound was irrigated thoroughly, and skin around the chamber was sutured to protect the wound and promote healing.

### Brain Region Segmentation and Alignment

The structural information of the marmoset brain was extracted from the raw MRI images using 3D slicer, a commercially available medical image processing software. Next, ANTs (Advanced Normalization Tools) were used to register the brain structure to the standard marmoset brain atlas (from the Brain/MINDS data portal), allowing for segmentation of each brain region.

### Locomotor Activity Measurement in a New Environment

Video recordings were carried out in the marmoset colony. The animals were transferred into a new cage (850 mm × 800 mm × 800 mm) with one wall transparent. The video recording started 5 min after the animal entered the cage, and its movement was recorded for 10 min by a video camera 1.5 m from the cage via the transparent wall. Trajectories were analyzed using a custom MATLAB program. To examine whether the implants would affect the physical mobility of marmosets, the hanging ratio, movement distance, and the count of jumps were calculated. The hanging ratio was defined as the ratio of the time the animal was hanging in the cage to the total time. The movement distances were the length of the trajectories during the recording. The count of jumps was counted manually using the recorded video.

### Recording Drinking Behavior

All animal experiments in this study were conducted in a custom‐built RF/EMI shielded chamber. In the drinking experiment, the experimental marmoset equipped with the device was placed in a specially designed cage that permitted unrestricted movement. A water bottle hung above the cage was wirelessly controlled by an experimenter to descend randomly. Upon noticing the descending bottle while in a state of thirst, the marmoset would swiftly approach the bottle to drink, with the bottle elevated 2 s after the drinking commenced. A frontal‐view camera was positioned 1.5 m from the cage to capture videos at a frame rate of 60 Hz during the drinking behavior. The neural signals and the video were recorded simultaneously.

### Recording Vocal Communication Behavior

In the vocal communication experiment, the experimental marmoset equipped with the device was placed into a cage, and it moved freely. A free‐field loudspeaker was placed 1 meter away from the cage. Two microphones were pointed at loud speaker and marmoset, respectively, in order that the sound could be recorded. Vocalization from other marmosets were played from the loudspeaker to promote marmoset vocalization. The neural signals and the audio signals were recorded simultaneously.

### Recording Vigilance Behavior to a Human Intruder

In the human intruder experiment, the experimental marmoset equipped with the device was brought to a custom‐built RF/EMI shielded chamber and placed into a box with only one side transparent. Then, one experimenter would enter the chamber and hide on the opaque side of the box. Next, the experimenter would wave a glove from the opaque to the transparent side to scare the marmoset and induce its vigilance behavior. Typically, upon seeing the glove, the marmoset would immediately jump backward. To prevent interference between vigilance trials, the next stimulus would not be performed until the marmoset had recovered from the scare (determined by visual inspection by the experimenter). The neural signals and the video were recorded simultaneously.

### Behavioral Annotation

First, for drinking behavior analysis, three consecutive phases were defined: Phase 1 (“Craving”) represents when the marmoset first notices the descending water bottle through visual or auditory cues; Phase 2 (“Seeking”) occurs while the marmoset approaching the bottle, typically by jumping from one side of the cage to the bottle; and Phase 3 (“Drinking”) begins when the marmoset obtains and consumes water by licking the bottle edge. One‐second periods were sampled for further analysis in each phase. “Craving”: 1 s before start the the movement. “Seeking”: 1 s before touching the bottle. “Drinking”: from 0.5 to 1.5 s after touching the bottle.

Second, for vocal communication analysis, marmoset phee calls were identified based on their distinctive spectrogram characteristics (8–20 kHz frequency range, 0.5–3 s duration). Using Raven Pro sound analysis software, raw audio files were converted into spectrograms and annotated calls by detecting these characteristic spectral patterns.

Third, for vigilance behavior, the experimenter elicited responses by waving a glove from the opaque to the transparent side of the cage to startle the marmoset. The behavioral onset was clearly defined as the exact video frame when the marmoset initiated its characteristic backward jump in reaction to the glove movement.

### Data Acquisition and Signal Pre‐Processing

Impedance was tested before recording to exclude channels with short or open circuit issues. 120‐channel ECoG signals were recorded at a sampling rate of 500 Hz. The analysis focused on low‐frequency components (1–200 Hz), as these have been consistently shown to encode the majority of behaviorally relevant neural information in ECoG recordings, particularly for natural behaviors. The signals were filtered off‐line using MATLAB software. Noise channels were manually removed, and trials with excess motion artifact were rejected. Subsequently, the signals from two electrode arrays (FC and TC) were mean re‐referenced separately to minimize the effect of volume conduction. All further analysis was performed using custom‐built MATLAB and Python code, as required for the analysis. Electrode locations for the multi‐area µECoG arrays were determined using pre‐operative MRI and brain atlas registration (Figure S11, Supporting Information).

### Spectrogram Visualization

Spectrograms were computed for each electrode to analyze the time‐frequency representation of multi‐area neural activities. The spectral information was extracted within a 500 ms analysis window (50 ms step‐size) using a multi‐taper time‐frequency analysis method implemented in Chronux function mtspectrumc.m (found at http://chronux.org/). The resultant spectrograms were normalized with respect to a baseline period. First, the resultant spectrogram was subtracted from its average baseline power spectral density, and then divided by this average baseline power spectral density. The normalized spectrograms were then averaged across trials around the behavior onset to quantify neural activations. The average power fluctuation for multiple frequency bands was calculated using the equation:

(1) $$ESNR\left(t\right)=\frac{1}{f2-f1}\sum_{f1}^{f2}\frac{Spect\left(t,f\right)-\frac{1}{\tau }\sum Spect_{\mathrm{baseline}}\left(\tau ,f\right)}{\frac{1}{\tau }\sum Spect_{\mathrm{baseline}}\left(\tau ,f\right)}$$

*f*1 *and* *f*2 are the lower and upper limits of the selected frequency band, respectively.

### Cortical State Space Analysis Using PCA

The pre‐processed neural signals from each electrode were band‐pass filtered into 6 frequency bands between 1 and 150 Hz (delta: 1–4 Hz, theta: 5–8 Hz, alpha: 9–14 Hz, beta: 15–30 Hz, low‐gamma: 31–80 Hz, high‐gamma: 81–150 Hz), and multi‐band energy features were calculated using the MATLAB function, bandpower.m. In the analysis of drinking neural signals across different phases, the multi‐band power was computed for all channels for each drinking trial and mean‐normalized it to the same baseline period. Then, principal component analysis (PCA) was used to transform the normalized multi‐band power features into a low‐dimensional subspace. In Figure 4f, the first two principal component scores were used to visualize high‐dimensional data. Each sample for diverse phases was represented by two vectors.

### Drinking Phases Classification Using SVM

A classification model with 5‐fold cross‐validation was constructed to decode drinking phases from multi‐band power features. First, PCA was used to compress the features matrix (training set) to a low‐dimensional subspace as explained in the previous section. Then, the resultant principal component scores were utilized to classify different phases using a linear support vector machine (SVM) model. The order of the resultant principal component was determined by the principal component scores that cumulatively explained 80% of the neural variance. In the analysis to evaluate the impact of spatial resolution and coverage on state‐space representation and decoding performance, multi‐band energy features were extracted from all electrodes in the selected regions for each case of spatially subsampled electrodes or different brain regions. Then, the SVD was repeated to obtain new principal component scores and trained the SVM model for decoding. Thirty rounds of 5‐fold cross‐validation were performed for each condition to ensure the accuracy of the results (Figure S14, Supporting Information).

### Pearson Correlation

In Figure S11 (Supporting Information), the Pearson correlation coefficients of ECoG signals from channels at different distances were calculated to verify the validity of the acquired signals. The correlation coefficient is calculated as follows:

(2) $$r=\frac{\sum \left(x-m_{x}\right)\left(y-m_{y}\right)}{\sqrt{\sum {\left(x-m_{x}\right)}^{2}\sum {\left(y-m_{y}\right)}^{2}}}$$

*m_x_* and *m_y_* are the means of ECoG signal x and signal y at different electrode positions.

### SNR

In Figure 7, SNR was defined as the dB ratio between the standard deviation of a range of noise amplitude against maximum absolute amplitude of the main signal of interest:

(3) $$SNR=20\times log_{10}\frac{ABS_{SignalMax}}{SD_{NoiseRange}}$$

Here, a baseline range (−2 s to −1 s before drinking onset) without notable activities was considered as noise range.

### Statistical Analysis

The neural signals were preprocessed using MATLAB software, including filtering, noise removal, and re‐referencing. Further analysis was performed using custom‐built MATLAB and Python code. Unless otherwise stated, all results are presented as mean ± SEM. For analyzing the average power across different phases of drinking behavior, the study used data from 72 trials of one animal and applied the Wilcoxon signed‐rank two‐sided test (*P < 0.05, **P < 0.01, ***P < 0.001).

## Conflict of Interest

The authors declare no conflict of interest.

## Author Contributions

H.L., X.C., J.L., and L.Z. contributed equally to this work. H.L., X.C., M.Z., X.L., and L.G. conceptualized the project. H.L., X.C., M.Z., X.L., J.L., L.Z., X.W., and C.X. designed and implemented the methodology. H.L., X.C., J.L., Q.L., M.X., Y.W., X.Z., and Y.W. carried out the investigations. H.L., J.L., H.L., L.Z., and N.D. created the visualizations. M.Z. acquired funding and managed the overall project. M.Z., X.L., L.G., L.L., C.Y., J.X., and K.Z. supervised the research. H.L., J.L., X.G., and T.S. wrote the original draft. H.L., X.C., J.L., L.Z., M.Z., and X.L. reviewed and edited the manuscript. All authors discussed the results and contributed to the final version of the manuscript.

## Supporting information



Supporting Information

Supplemental Movie 1

Supplemental Movie 2

Supplemental Movie 3

## Data Availability

The data that support the findings of this study are available from the corresponding author upon reasonable request.
