# Supplementary material for: Deciphering Neural Mechanisms Underlying Marmoset Dynamic Natural Behaviors Using a Miniaturized Wireless Large‐Scale Coverage Neural Recorder
Source: Adv Sci (Weinh). 2025 Oct 27;13(2):e07110. doi: 10.1002/advs.202507110 (PMC12786299; doi:10.1002/advs.202507110)
Supplement: Supplementary file 1 — Supporting Information [file ADVS-13-e07110-s002.docx]

Supporting Information

**Title**

Deciphering Neural Mechanisms Underlying Marmoset Dynamic Natural Behaviors Using a Miniaturized Wireless Large-Scale Coverage Neural Recorder

*Hongru Liu, Xinyuan Cao, Jiyong Li, Lingyi Zheng, Jingwei Li, Qianbing Li, Min Xie, Huimin Li, Xiaolong Wang, Yuyu Wu, Xiangyu Zhang, Yizheng Wang, Xize Gao, Tiancheng Sheng, Nianzhen Du, Chengao Xu, Kai Zhou, Jing Xu, Changxiang Yan, Lianqing Liu, Lixia Gao*, Xinjian Li* and Mingjun Zhang**

**The file includes:**

Supplementary Figure S1 to S19.

Supplementary Table S1 to S2.

**Other Supporting Information for this manuscript includes:**

Supplementary Videos S1 to S3.

**Supplementary Figures and Captions**


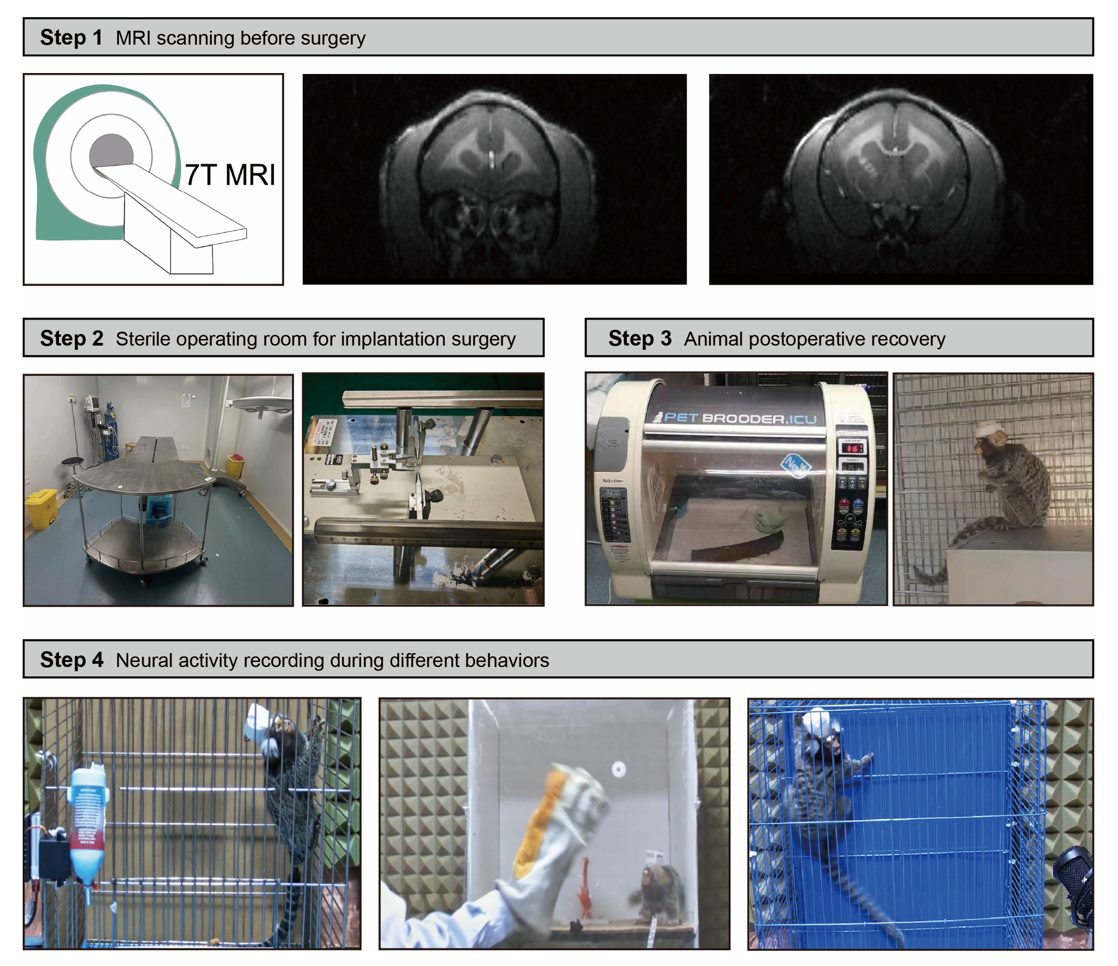


**Figure S1. Surgical environment and flow of marmosets**

**Step 1**: 7T MRI scanning was performed before surgery for calculating the target brain region coordinates. **Step 2**: The surgery was conducted in a sterile operating room. **Step 3**: Animals recovered in the incubator and were transferred to the homecage after temperature stabilization. **Step 4**: Photographs of three natural behaviors in this study (drinking, vigilance to the human intruder, and vocal communication). All experiments were conducted in a custom-built RF/EMI shielded room.


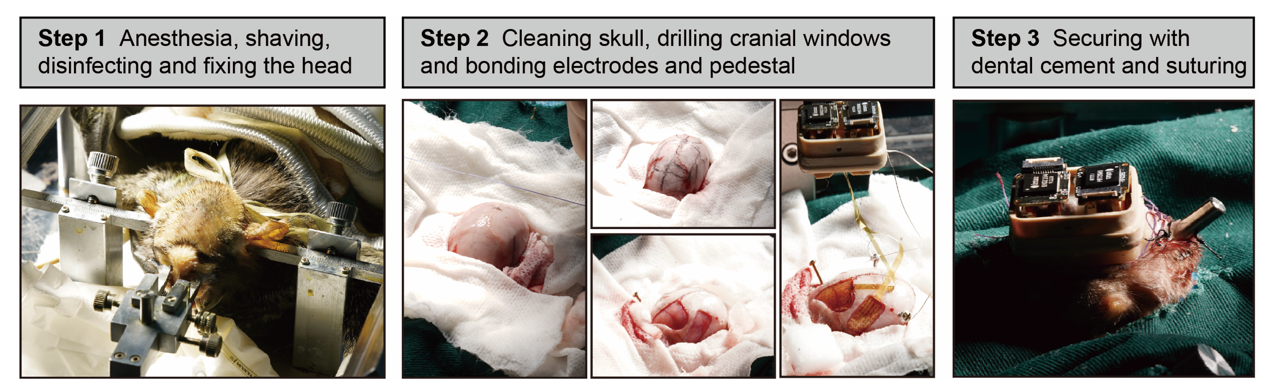


**Figure S2. Detailed surgery procedure of the implantation**

**Step 1**: Animal was anesthetized, shaved, sterilized and fixed to the stereoscope. **Step 2**: Cranial window was drilled after cleaning the skull and electrodes were attached. Chamber was bonded on the skull. **Step 3**: Dental cement was used to secured the system and wound was sutured.


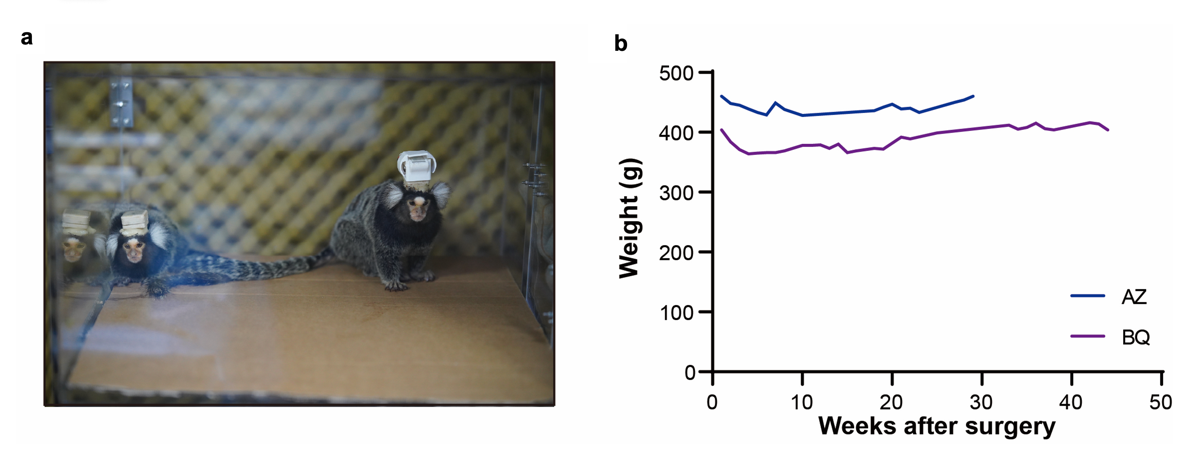


**Figure S3. Surgical environment and flow of marmosets**

**a,** Photograph of the marmosets’ condition after recovery. **b,** Weight curve of marmosets after surgery.

**
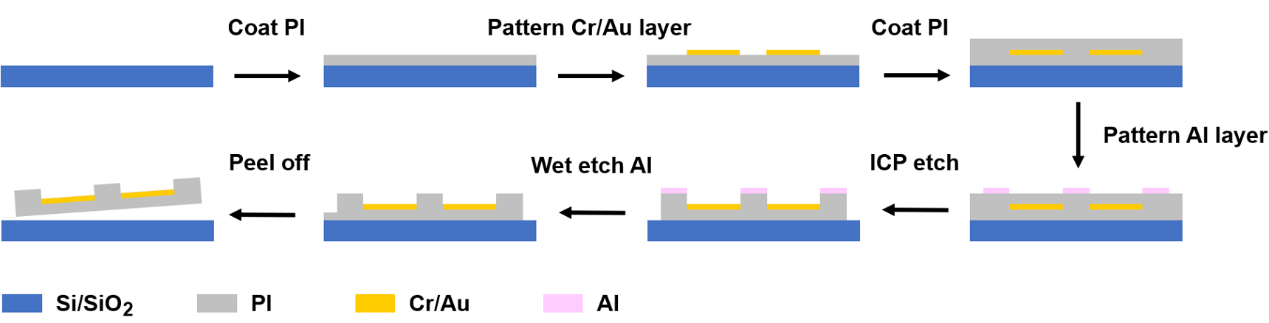
**

**Figure S4. Schematic diagram of the fabrication process for the custom-designed high-density μECoG arrays**

**
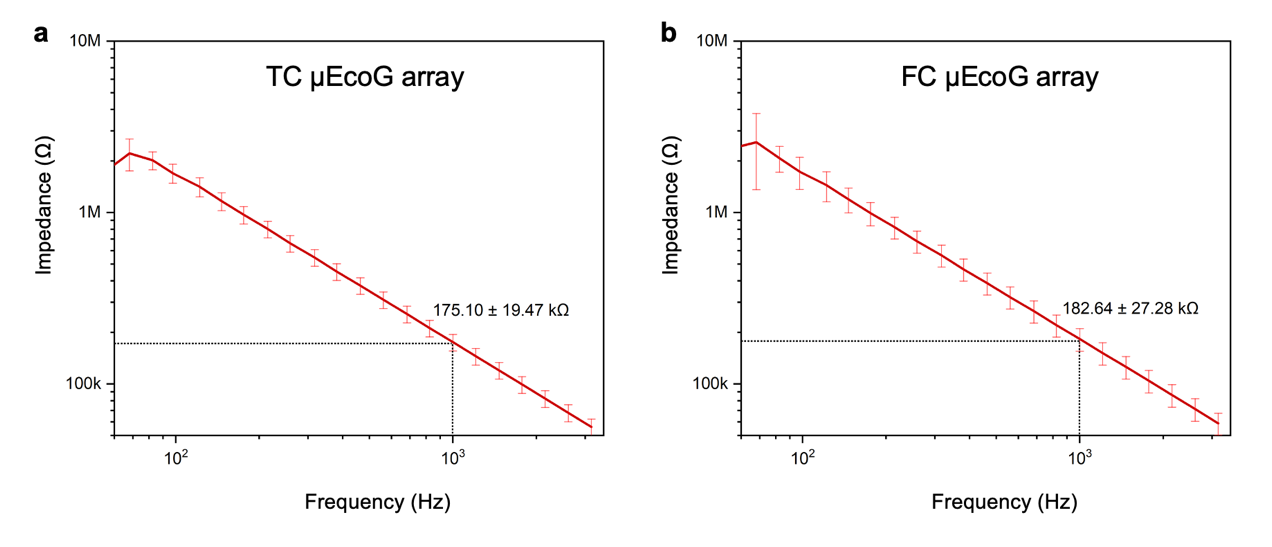
**

**Figure S5. EIS magnitude of the custom-designed high-density μECoG arrays**

**a,** EIS magnitude of TC μECoG array (mean ± s.d., n=51 electrodes). **b,** EIS magnitude of FC μECoG array (mean ± s.d., n=47 electrodes).


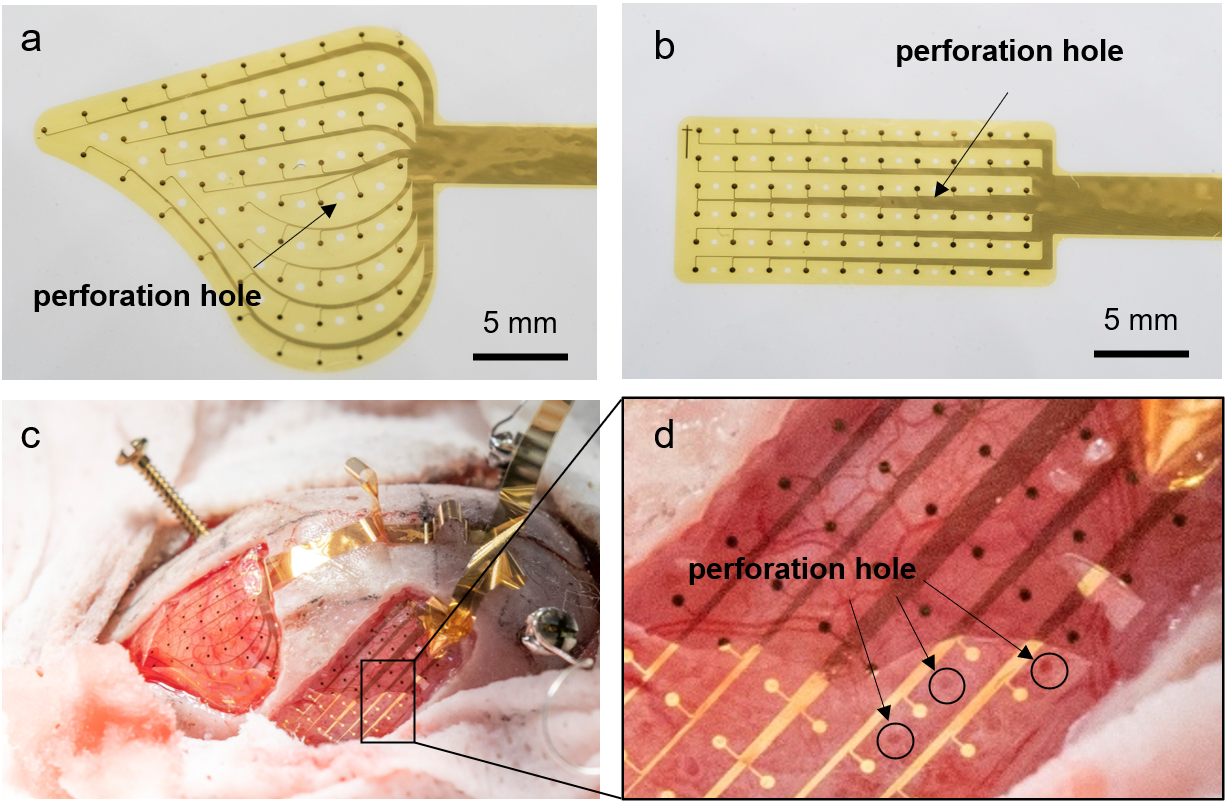


**Figure S6. Perforation hold design of the custom-designed μECoG arrays**

**a**, **b**, Photographs of the implanted portions of FC μECoG arrays (**a**) and TC μECoG arrays (**b**). **c**, Intraoperative photograph of the custom-designed μECoG arrays. **d**, Zoomed-in view of the region marked by the black box in panel (**c**). The black circles mark the perforation holes.


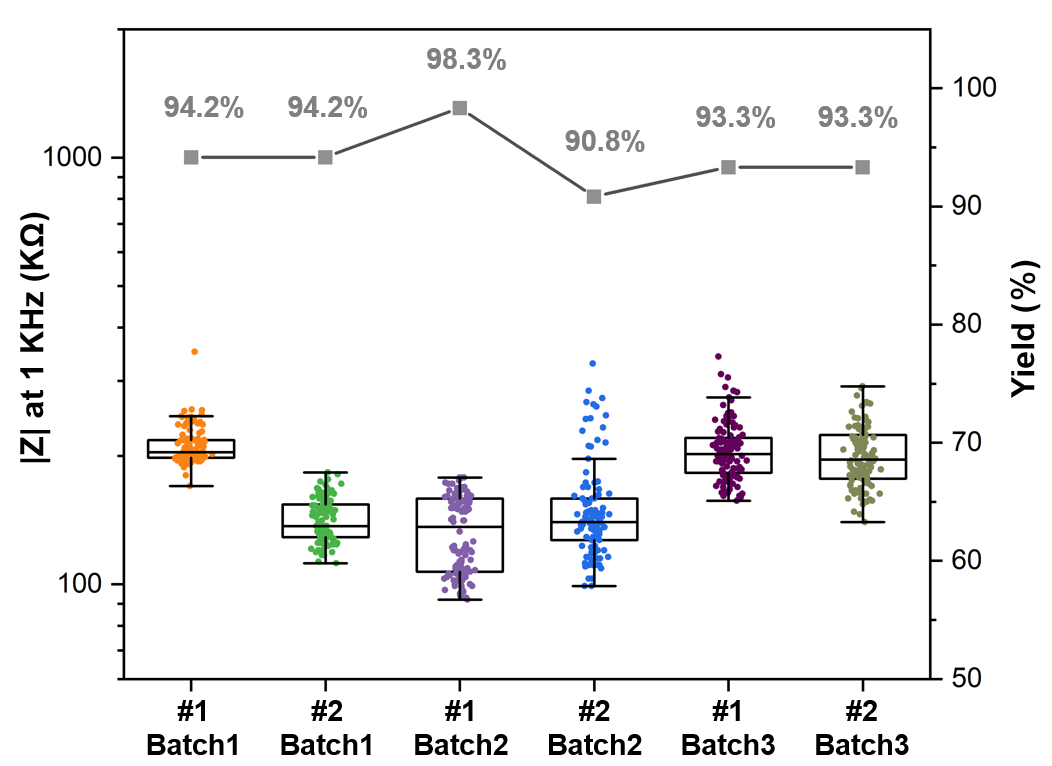


**Figure S7. Yield and manufacturing stability test of the custom-designed μECoG arrays**

The box plots show median and quartile range of the impedance values of each set of μECoG array, with whiskers denoting 1.5× the interquartile range. Individual data points are overlaid on the box plots. The gray dash at the top shows the yield of each set of μECoG array.


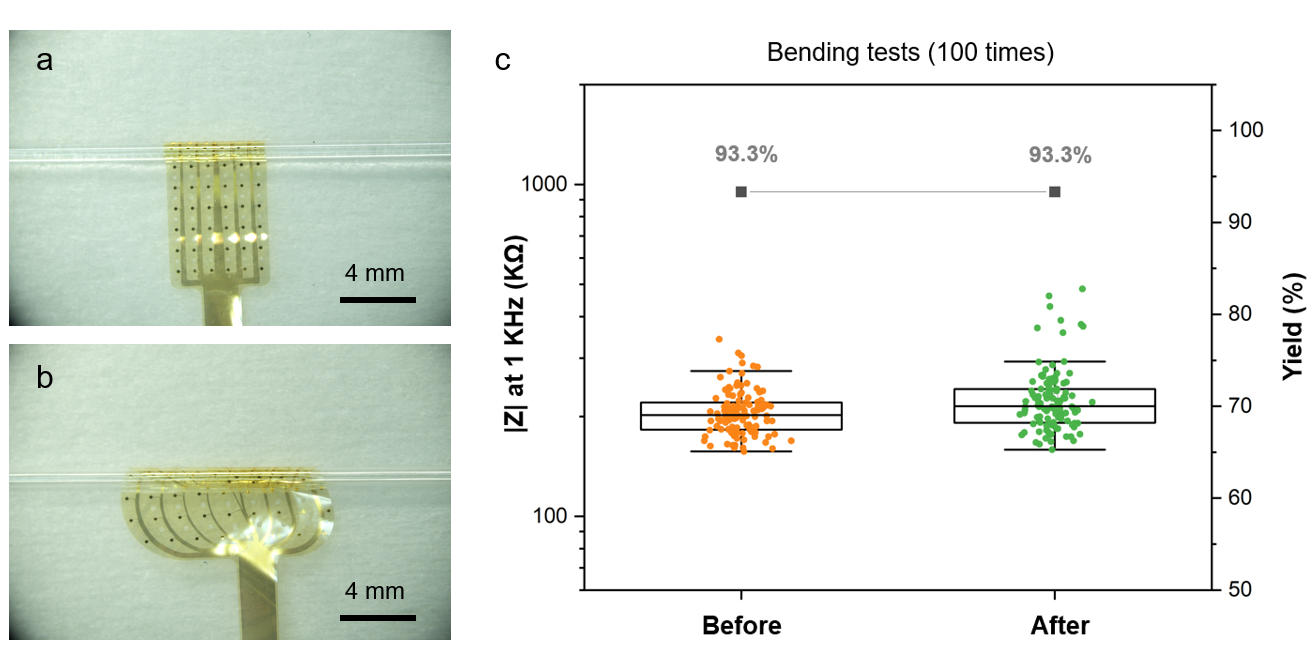


**Figure S8. Bending test of the custom-designed μECoG array**

**a**, **b**, Photographs of the setup of bending test. TC μECoG array (**a**) and FC μECoG array (**b**) were manually wrapped around a glass rod (1.2mm in diameter) for 100 times, respectively. **c**, Impedance measurements before and after the bending test. The box plots show median and quartile range of the impedance values of one set of μECoG array, with whiskers denoting 1.5× the interquartile range. Individual data points are overlaid on the box plots. The gray dash at the top shows the yield of the set of μECoG array before and after the bending test.


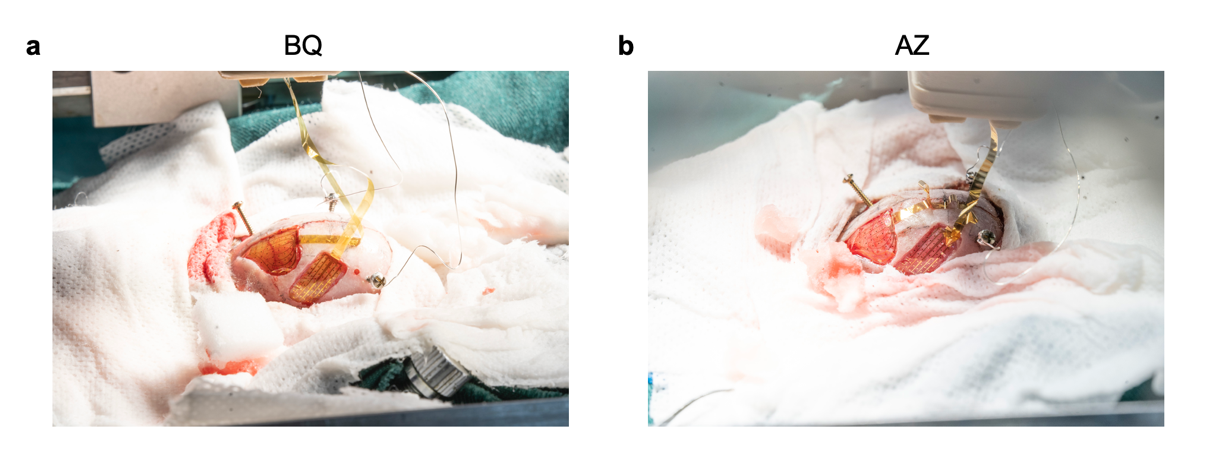


**Figure S9. Photographs of BMI implantation surgery in two marmosets**

Two custom-designed, high-density flexible 60-electrode μECoG arrays implanted epidurally into a marmoset brain through minimally invasive surgery.


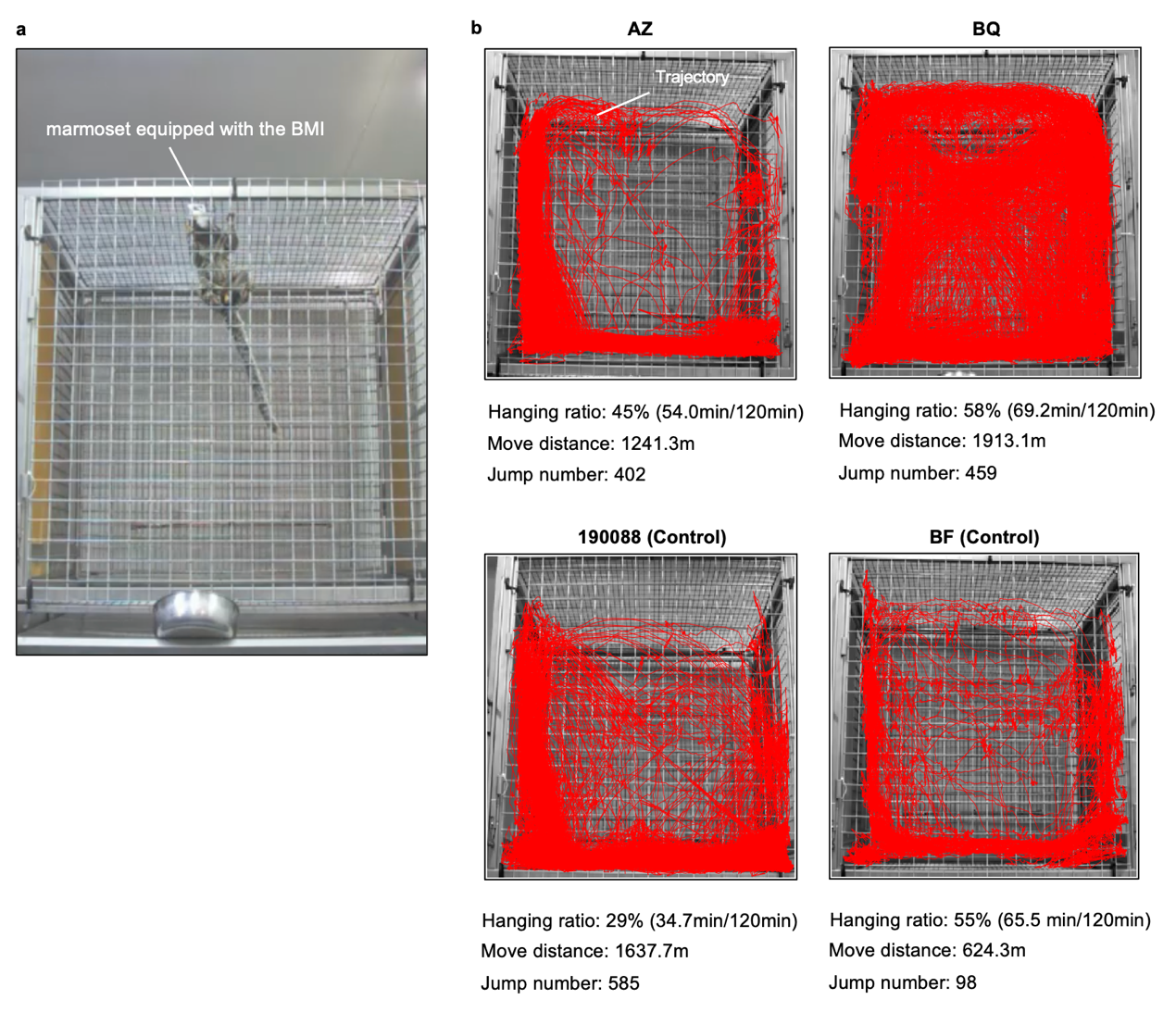


**Figure S10. Locomotor activity measurement in an open-field cage for four marmosets**

To examine whether wearing the system would affect the physical mobility of marmosets (with implanted electrodes: AZ, BQ; control group: 190088, BF), we calculated the hanging ratio, distance moved, and the number of jumps. The hanging ratio was defined as the ratio of the time the animal was hanging in the cage to the total time. The distances moved were the length of the trajectories during the recording. The number of jumps was counted manually using the recorded video.

**
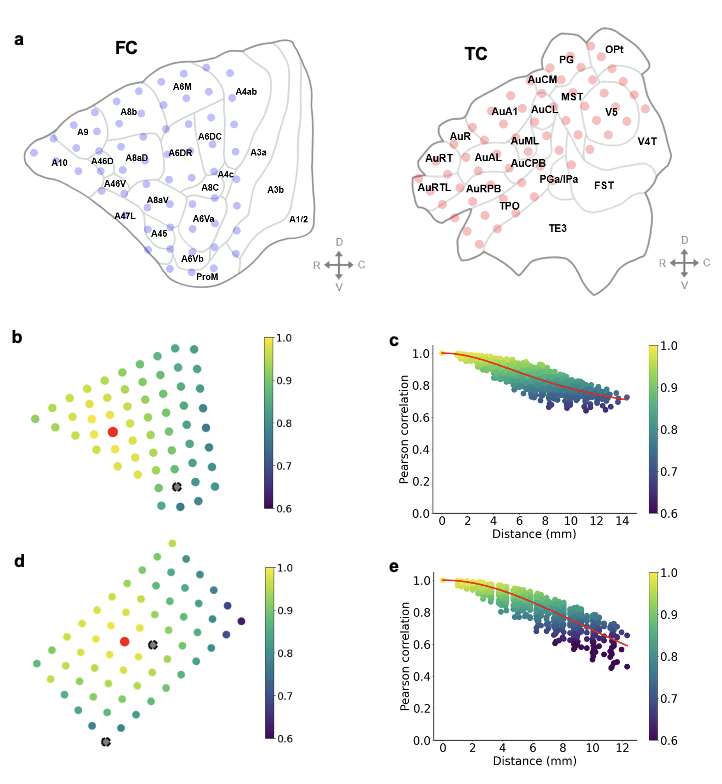
**

**Figure S11. The electrode locations and inter-electrode correlation**

**a**, The electrode locations of FC and TC μECoG array. The brain area structures were obtained using pre-operative MRI imaging and brain atlas registration. This schematic diagram was drawn based on the planned locations of preoperative electrode implantation. **b**, **d**, ECoG signals (1-200Hz) spatial spread for FC and TC. The red circles indicate the channels selected for calculating the correlation. The gray circles with a black dashed outline indicate that the channel fails to record neural signals. **c**, **e**, Inter-electrode Pearson correlation decreased with increased electrode spacing for FC and TC. The red curve illustrates the result of Lorentzian fitting applied to the scatter points.

**
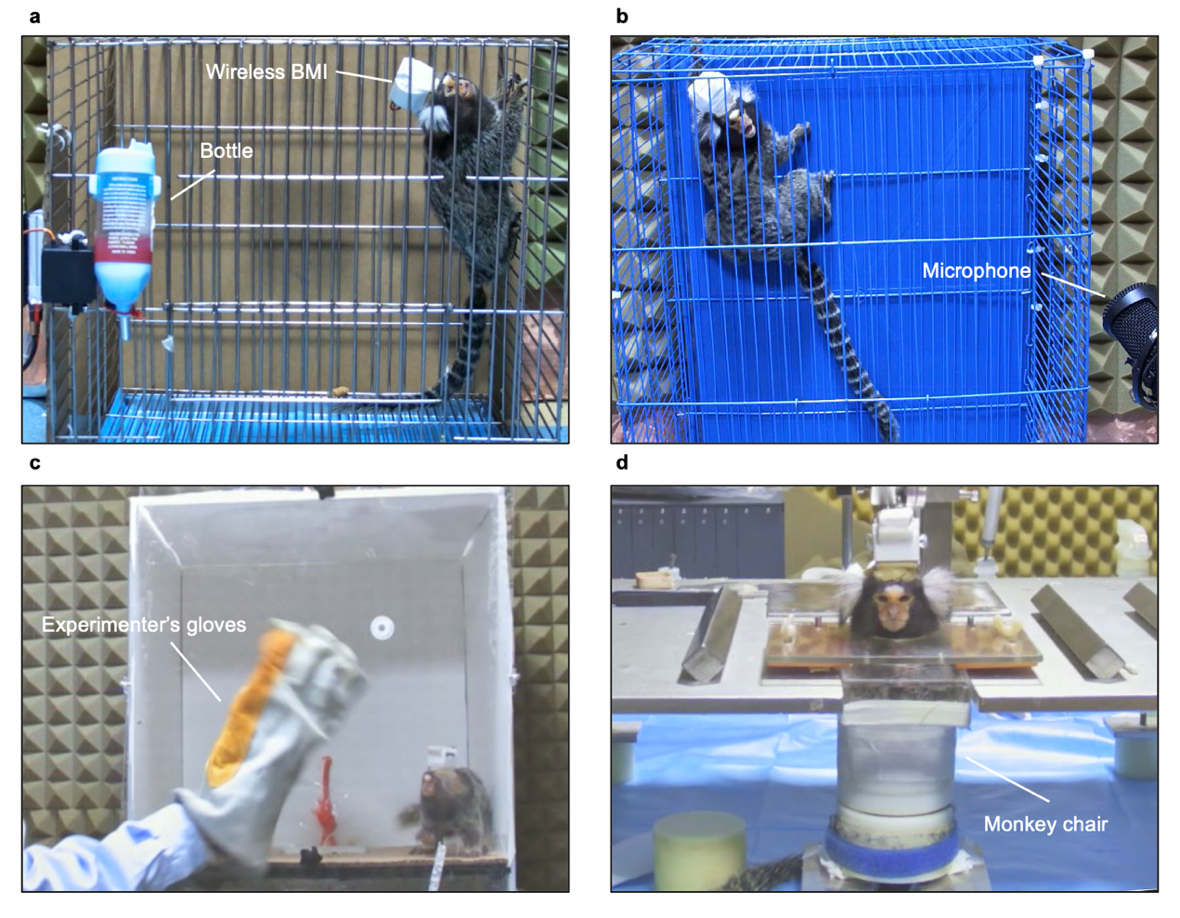
**

**Figure S12. Photographs of diverse behaviors**

All animal experiments in this study were conducted in a custom-built RF/EMI shielded chamber. **a,** The drinking experiment. The marmoset was placed in a specially designed cage that permitted unrestricted movement. A water bottle hung above the cage was wirelessly controlled by an experimenter to descend randomly. Upon noticing the descending bottle while in a state of thirst, the marmoset would swiftly approach the bottle to drink, with the bottle elevated 2 seconds after the drinking commenced. **b,** The vocal communication experiment. The marmoset was placed into a cage. A free-field loudspeaker was placed 1 meter away from the cage. Two microphones were pointed at loud speaker and marmoset respectively. Vocalization from other marmosets were played from the loudspeaker to promote marmoset vocalization. **c**, The vigilance to the human intruder. The marmoset was placed into a box with only one side transparent. The experimenter would wave a glove from the opaque to the transparent side to scare the marmoset and induce its vigilance behavior. Typically, upon seeing the glove, the marmoset would immediately jump backward. **d**, The resting state recording. The marmoset was restrained in a monkey chair to prevent interference with neural signals from scratching or movement.


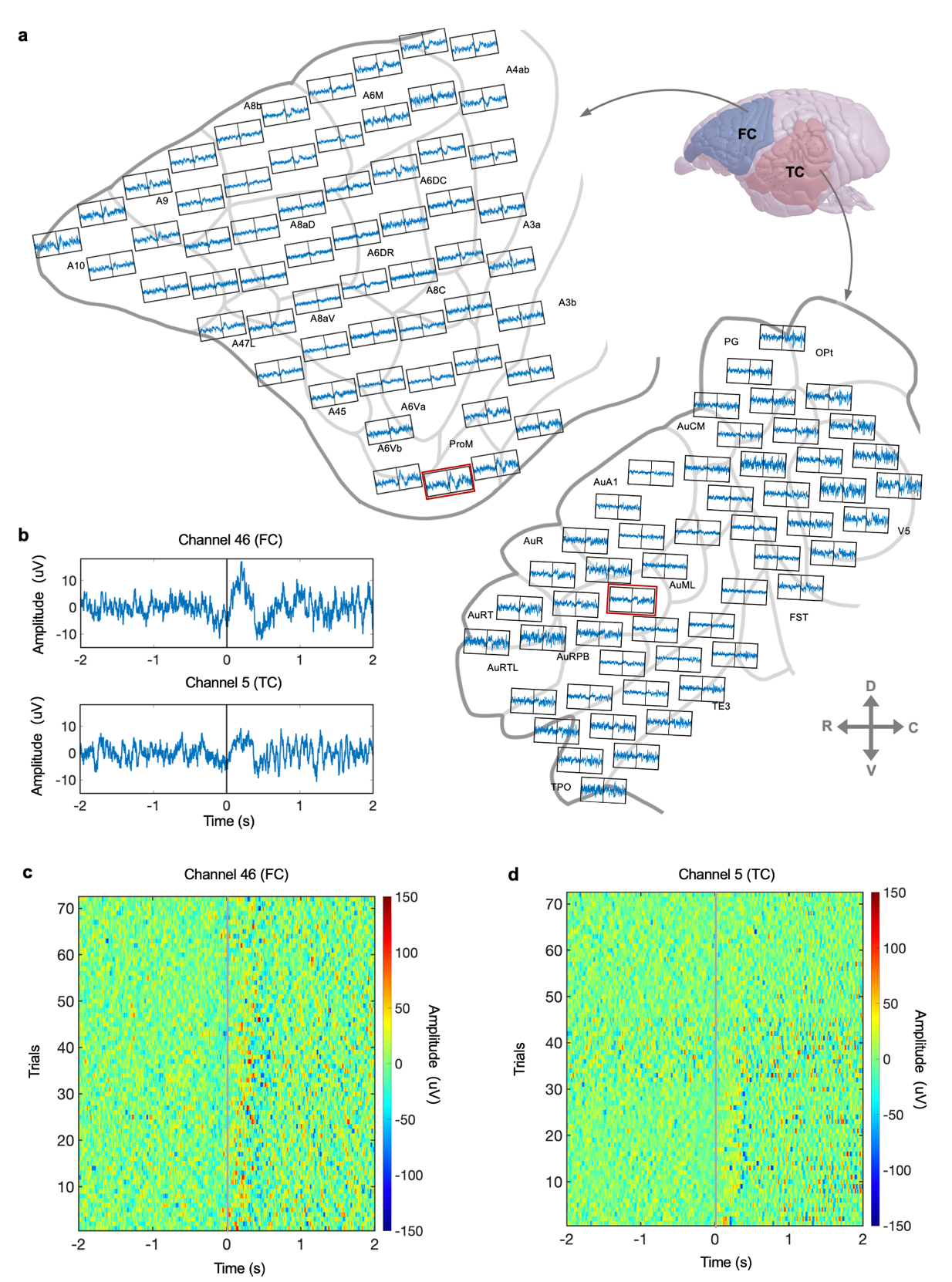


**Figure S13. Event-related potentials in FC and TC during drinking behavior**

**a,** Event-related potentials for each electrode from the FC and TC μECoG arrays during drinking behavior (averaged across all drinking trials, n=72). ECoG signals were aligned to the drinking onset. Vertical line: time zero of the drinking onset. **b**, Event-related potentials of two example electrodes (red border in (**a**)) demonstrated rapid increase following the drinking onset. **c, d,** Single-trial response from the electrodes same as (**b**).


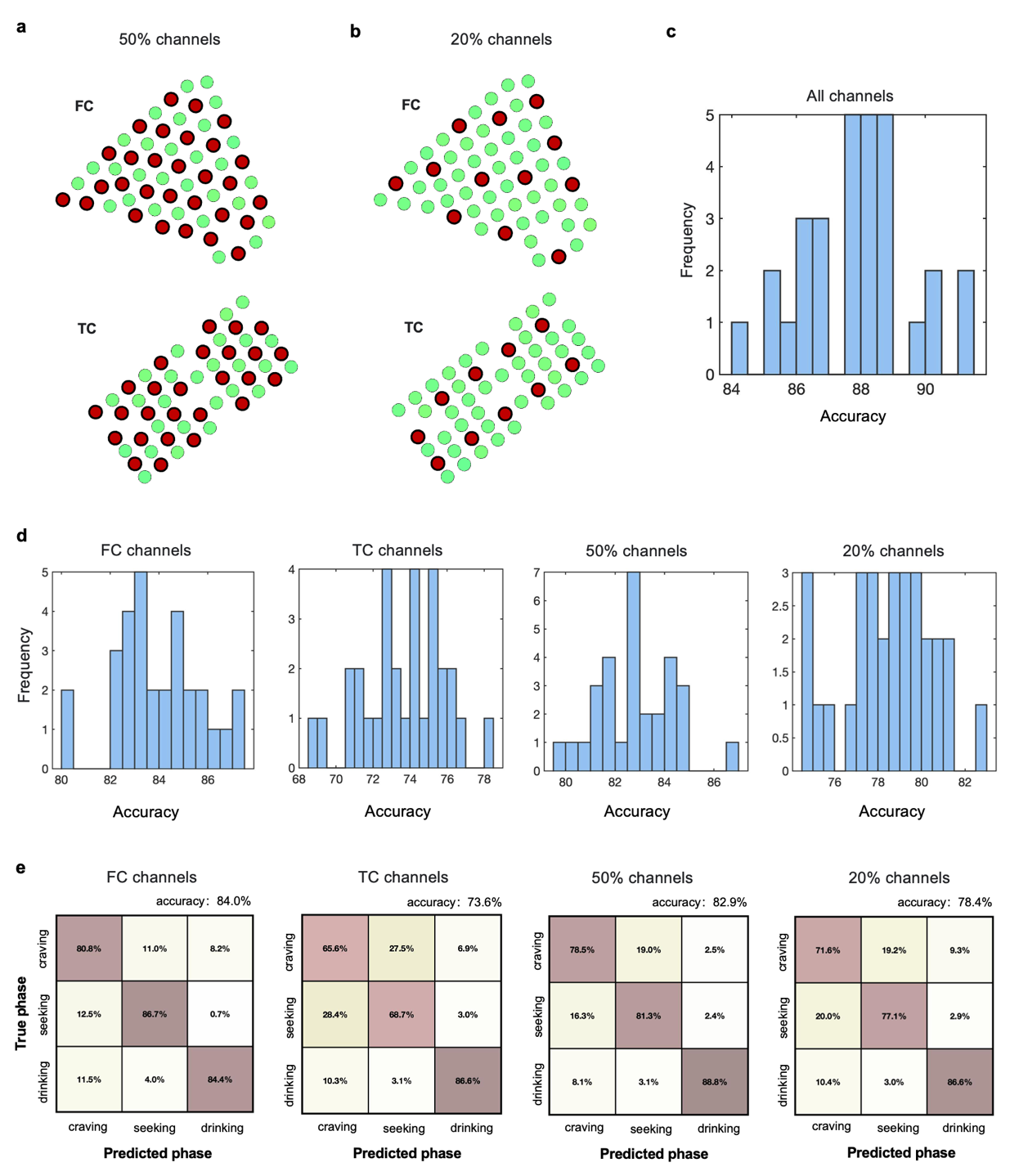


**Figure S14. Decoding performance under different spatial resolutions and specific regions**

**a, b,** Selected sites of FC and TC arrays under 50% and 20% channel conditions. The red circles indicate the selected channels, while the green circles represent the original channels. The blank areas indicate the channels that were excluded. **c**, Distribution of decoding accuracy for the three drinking phases using SVM, based on thirty rounds of 5-fold cross-validation with all electrodes. **d**, Distribution of decoding accuracies (30 rounds of 5-fold cross-validation) for different sub-sampling strategies and the number of brain regions. **e,** Confusion matrix for the three phases under different sub-sampling strategies and the number of brain regions.


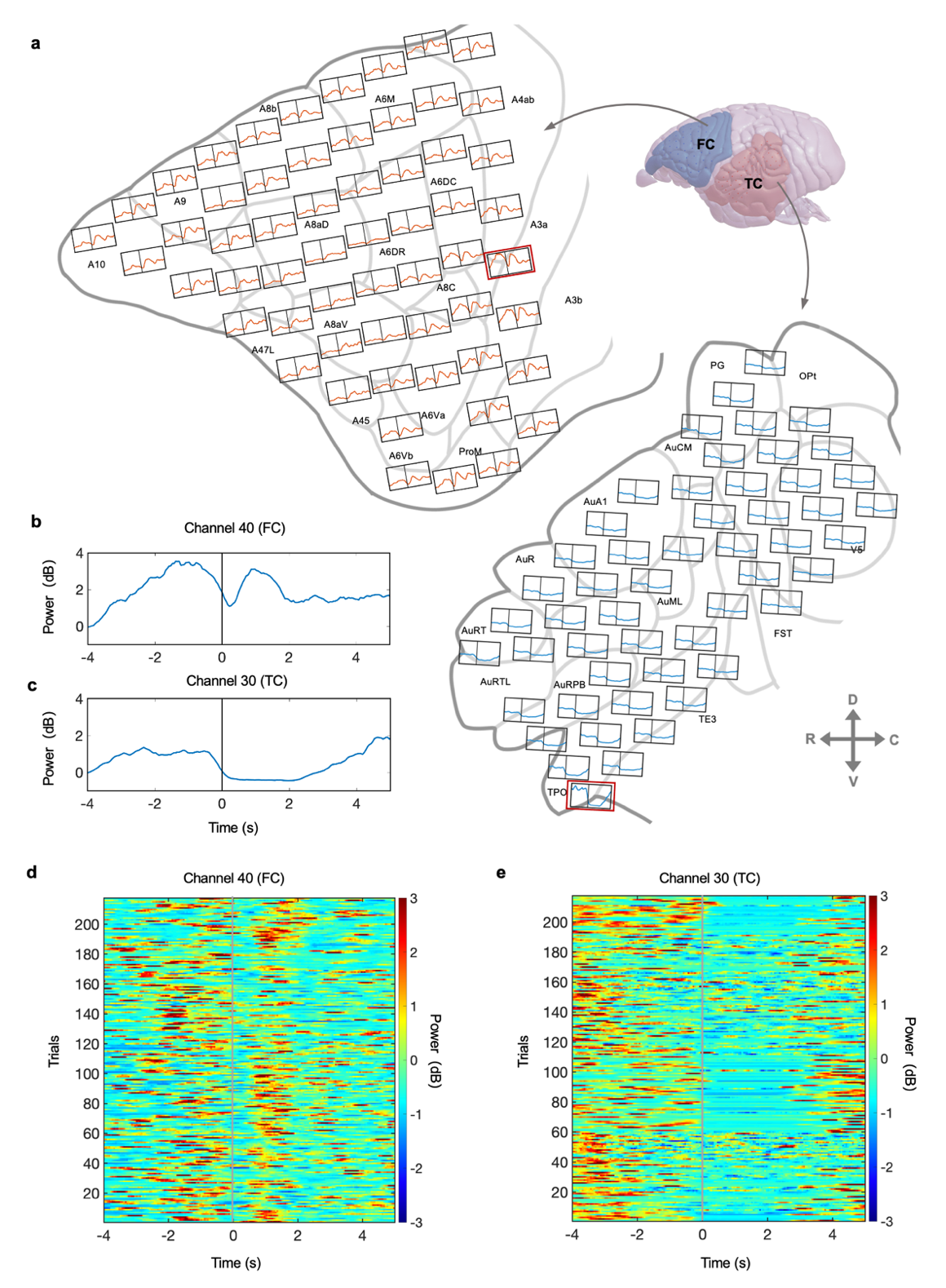


**Figure S15. Alpha and gamma activities in FC and TC during phee call behavior**

**a,** Alpha-band (8-13Hz) power from the FC array and gamma-band (30-80Hz) power from the TC array compared to the baseline window ([-4.5, -4] sec) prior to the vocal onset (averaged across all trials, n=217). Signals were aligned to the vocal onset. Vertical line: time zero of the vocal onset. **b**, **c**, Average alpha-band power of an example electrode from FC and TC (red border in (**a**)). **d,** Single-trial alpha power activation from the electrode same as (**b**). **e,** Single-trial gamma power suppression from the electrode same as (**c**).

**
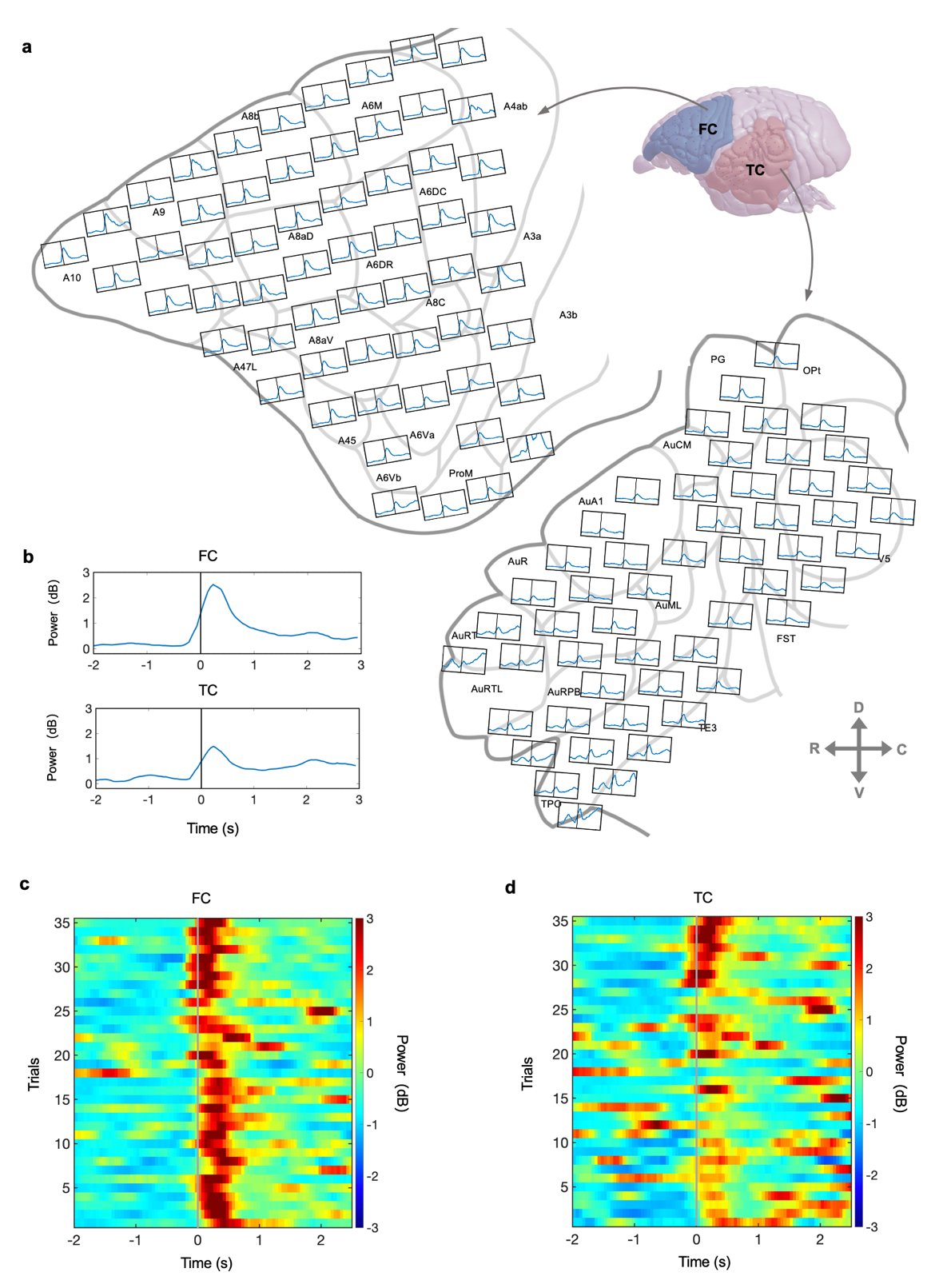
**

**Figure S16. High-gamma activity in FC and TC during the vigilance to the human intruder**

**a,** High-gama band (80-150Hz) power for each electrode from the FC and TC μECoG arrays compared to the baseline window ([-2.5, -2] sec) prior to the jump onset (averaged across all trials, n=35). ECoG signals were aligned to the jump onset. Vertical line: time zero of the jump onset. **b**, Average high-gamma band power of the all FC and TC channels. High-gamma activation in FC was stronger than that in TC. **c,** **d,** Single-trial high-gamma band power activation for FC and TC (averaged across all FC and TC channels).


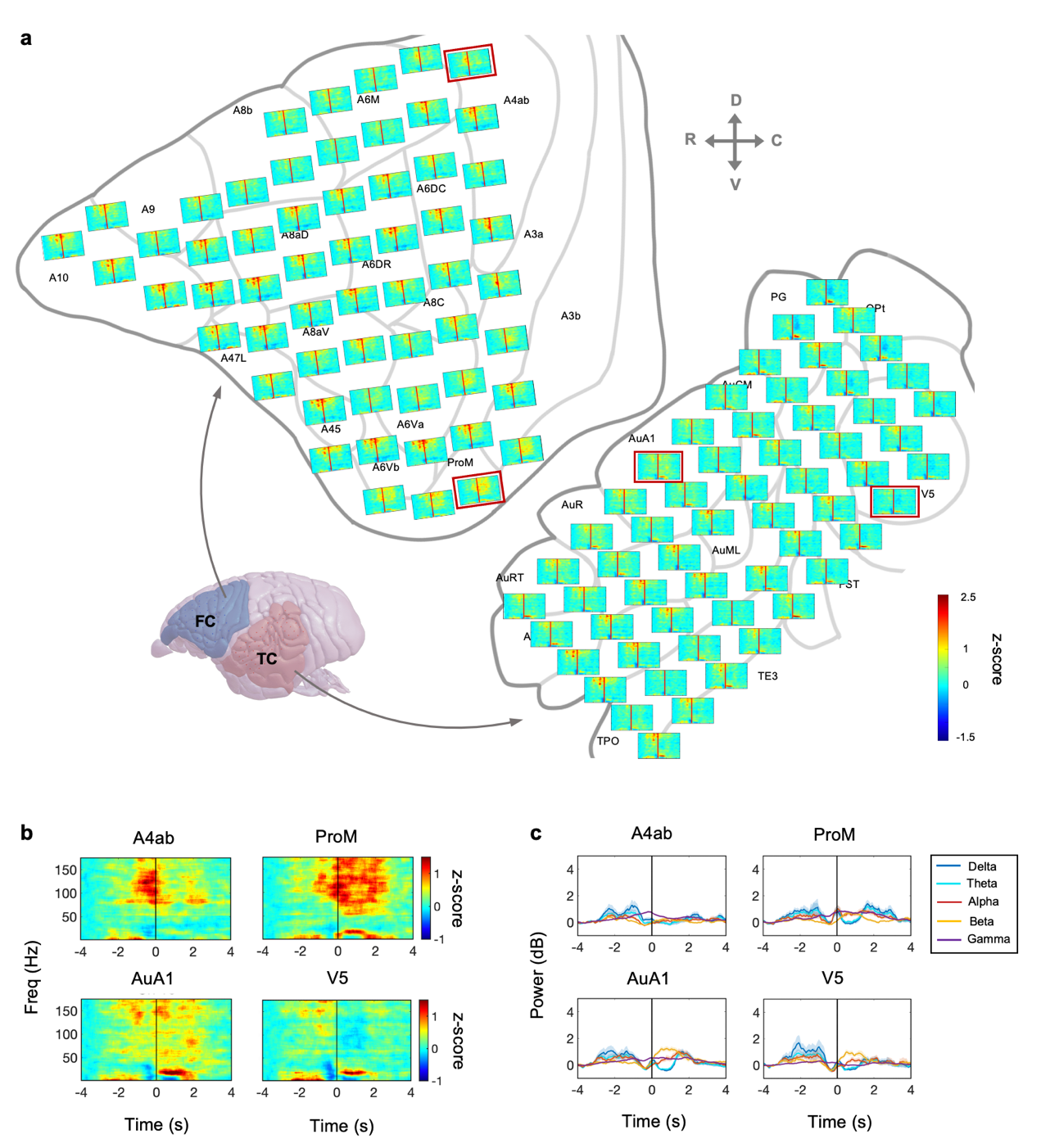


**Figure S17. Distinct highly resolved neural activities during drinking in the second marmoset AZ**

**a**, Schematic image showing the trial-averaged spectrograms of drinking induction neural activity using both FC and TC μECoG arrays (n = 39 trials). 5 damaged and high-impedance channels were discarded and displayed as empty. **b**, Spectrograms of four sampled electrodes from 4 separated brain regions (A4ab, ProM, AuA1, V5, red border in (**a**)). Vertical line: the drinking onset. **e**, The exampled neural response of A4ab, ProM, AuA1, V5 during drinking at five frequency bands. The power from each frequency band was aligned following the drinking onset and averaged across trials (mean ± SEM). Blue: Delta band, 1-4 Hz; Cyan:Theta band, 5-8 Hz; Red: Alpha band, 9-14 Hz; Yellow: Beta band, 15-30 Hz; Purple: Gamma band, 31-180 Hz.


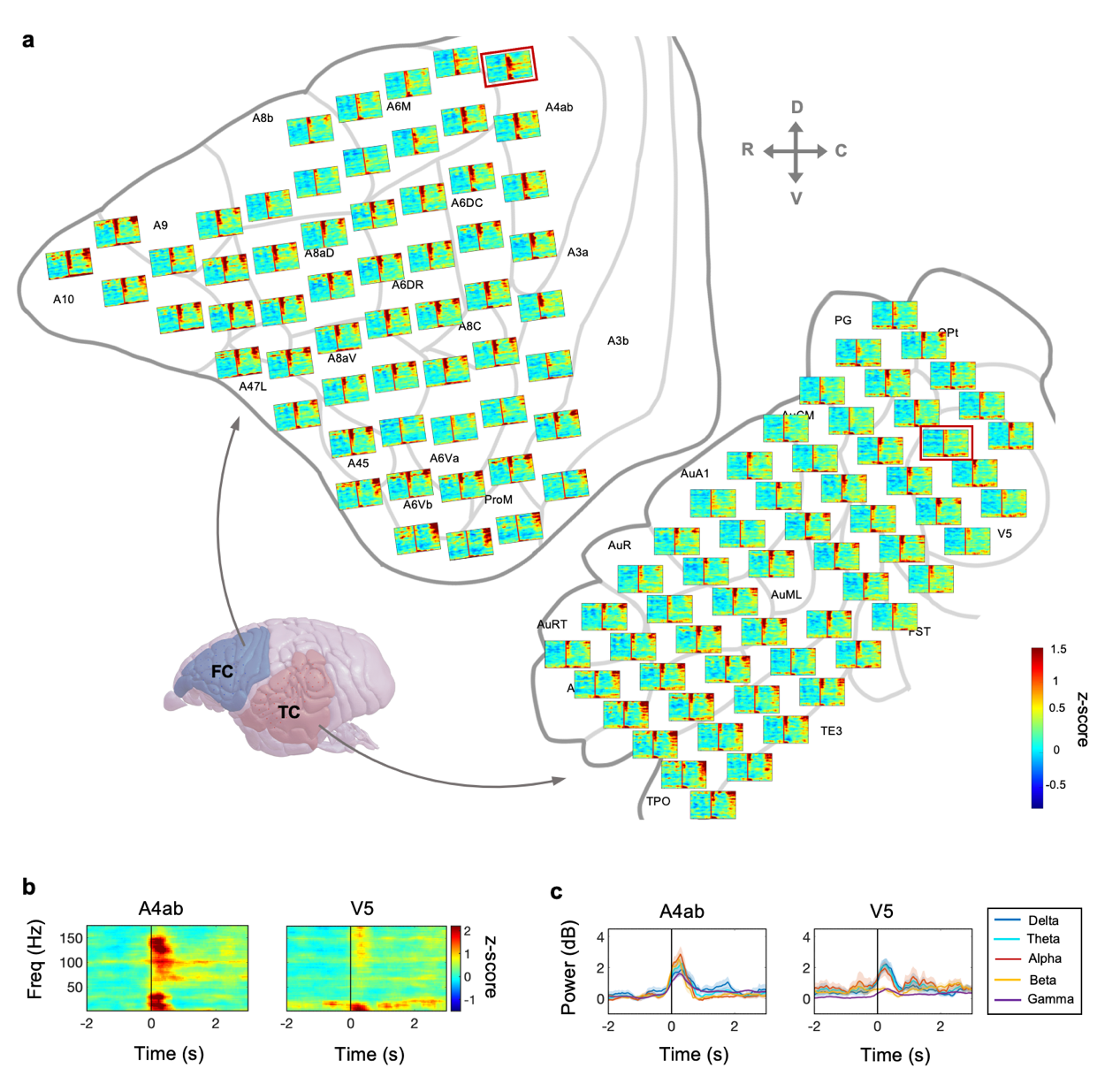


**Figure S18. Transient high-gamma activation during vigilance to human intruders in marmoset AZ**

**a**, Schematic image showing the trial-averaged spectrograms using μECoG arrays on FC and TC during the marmoset displaying of vigilance to human intruders (n=40 trials). **b,** Spectrograms of two sampled electrodes from FC (A4ab) and TC (V5). Vertical line, the marmoset shows the escape jumping behavior. **c,** The trial-averaged neural response of FC (A4ab) and TC (V5) during the marmoset shows the escape behavior at delta, theta, alpha, beta and gamma bands (n=40 trials). Blue: Delta band, 1-4 Hz; Cyan: Theta band, 5-8 Hz; Red: Alpha band, 9-14 Hz; Yellow: Beta band, 15-30 Hz; Purple: Gamma band, 31-180 Hz.


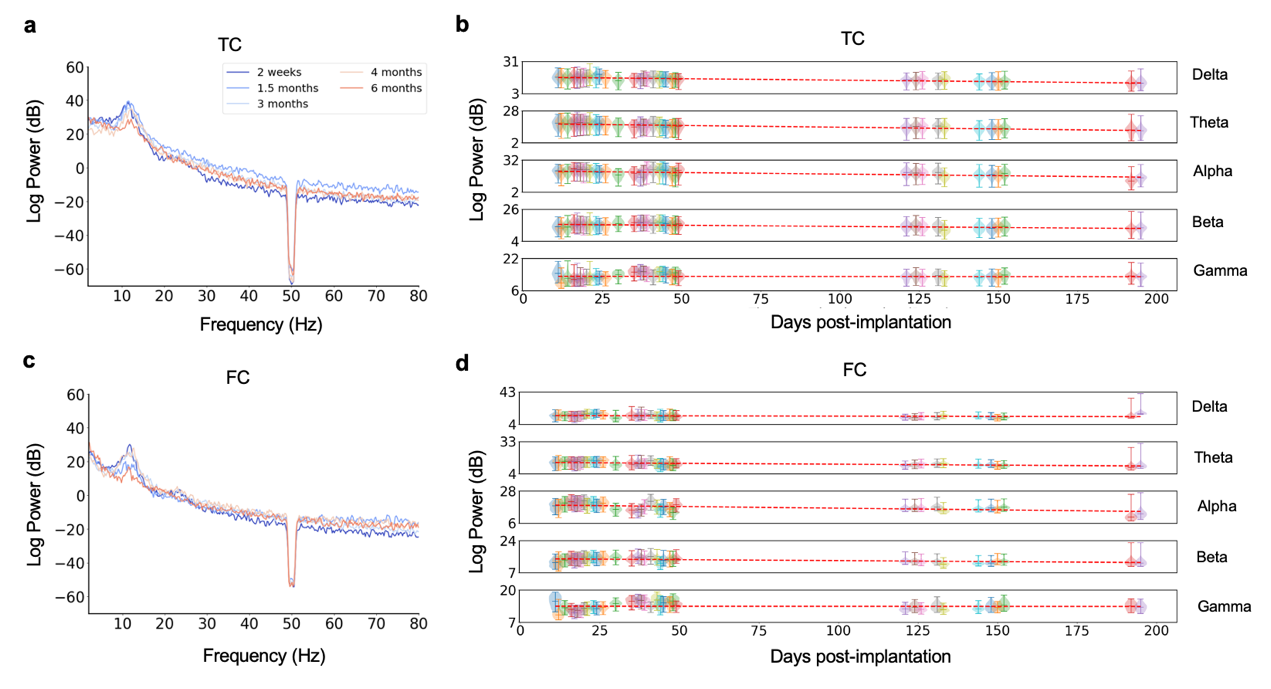


**Figure S19. Long-term stability of the BMI in marmoset AZ**

**a, c**, Power spectral density of signals recorded during the resting state in two sampled electrodes from the TC and PFC μECoG arrays over 6 months. **b, d**, Power variations of four typical band signals (delta: 1-4 Hz, theta: 5-8 Hz, alpha: 9-14 Hz, beta: 15-30 Hz, gamma: 31-150 Hz) recorded from all TC and FC channels in resting state over 6 months.

**Supplementary Tables**

| Institution | Electrode Type | Recording Channels | System Type | Recording Capability  From Freely Moving Animals | Reported Implantation Duration |
| --- | --- | --- | --- | --- | --- |
| RIKEN Brain Science Institute | Whole-brain ECoG | 64 | Wired | **×** | 4 months |
| Max Planck Institute | Silicon Probe | 192 | Wired | **×** | 35 months |
| Johns Hopkins University | Microwire Electrode | 16 | Wireless | **√** | 260 days |
| University of Chicago | Utah Array | 96 | Wireless | **√** | 3 years |
| Our device | High-density μECoG | 120 | Wireless | **√** | 16 months  (Ongoing, started by March 13th, 2024) |

**Table S1. List of neural signal recoding systems used in marmoset studies**

| Character | Marmoset BQ | Marmoset AZ |
| --- | --- | --- |
| Sex | Female | Male |
| Body weight before surgery (g) | 410 | 440 |
| Current body weight (g) | 405 | 460 |
| Implantation time | March 13th, 2024 | June 28th, 2024 |
| Coordinates of implanation TC cephalic from interaural line (mm) | 11 | 11 |
| Coordinates of implanation FC cephalic from interaural line (mm) | 18.5 | 18 |

**Table S2. List of animals and procedures**
